# A Single Dynamic Metabolic Model Can Describe mAb Producing CHO Cell Batch and Fed-Batch Cultures on Different Culture Media

**DOI:** 10.1371/journal.pone.0136815

**Published:** 2015-09-02

**Authors:** Julien Robitaille, Jingkui Chen, Mario Jolicoeur

**Affiliations:** Research Laboratory in Applied Metabolic Engineering, Department of Chemical Engineering, École Polytechnique de Montréal, C.P. 6079, Centre-ville Station, Montreal (Quebec), Canada; Universidad de La Laguna, SPAIN

## Abstract

CHO cell culture high productivity relies on optimized culture medium management under fed-batch or perfused chemostat strategies enabling high cell densities. In this work, a dynamic metabolic model for CHO cells was further developed, calibrated and challenged using datasets obtained under four different culture conditions, including two batch and two fed-batch cultures comparing two different culture media. The recombinant CHO-DXB11 cell line producing the EG2-hFc monoclonal antibody was studied. Quantification of extracellular substrates and metabolites concentration, viable cell density, monoclonal antibody concentration and intracellular concentration of metabolite intermediates of glycolysis, pentose-phosphate and TCA cycle, as well as of energetic nucleotides, were obtained for model calibration. Results suggest that a single model structure with a single set of kinetic parameter values is efficient at simulating viable cell behavior in all cases under study, estimating the time course of measured and non-measured intracellular and extracellular metabolites. Model simulations also allowed performing dynamic metabolic flux analysis, showing that the culture media and the fed-batch strategies tested had little impact on flux distribution. This work thus paves the way to an *in silico* platform allowing to assess the performance of different culture media and fed-batch strategies.

## Introduction

Monoclonal antibody (mAb) production at industrial level has reached, over the last decades, a 100-fold increase of the titers with up to 10 g L^-1^ [1]. This significant improvement can be explained by the ability to maintain very high cell concentrations (>10^7^ cells mL^-1^) at high viability for an extended period of time (i.e. weeks), a level of achievement resulting from cell engineering works, and the optimization of culture media composition coupled with efficient fed-batch strategies [2]. Serum-free media are complex to elaborate, because of a high number of essential and non-essential nutrients, as well as growth factors cocktail stimulating cell growth, viability and productivity in a recombinant product. Statistical methods, within a design of experiment approach, have been widely used to ameliorate culture media composition, both for screening active factors [3,4] and for optimizing components concentration [5,6]. The integration of the knowledge acquired over the past decades on optimal media composition allowed to extend culture duration in time-based fed-batch strategies overcoming culture media limitations. Efficient fed-batch strategies are thus designed to maximize growth and/or cell viability, while limiting the production of metabolic wastes, such as lactate and ammonia, which inhibit cell growth and affect the mAb product production and quality [7]. Indeed, various fed-batch approaches have been proposed such as from the stoichiometric feeding of nutrients with their consumption by the cells [8], medium feeding determined from a statistical design [9] or from the online control of glucose and glutamine at low levels to favour a more efficient metabolism [10,11]. Those approaches require a large number of data sets and thus rely mostly on time-consuming experimentation schedules that are determined either intuitively or randomly. An approach based on comprehensive mechanistic relationships could, however, help understanding how cells interact with their culture medium.

Metabolic flux analysis (MFA) and flux balance analysis (FBA) studies have been conducted on CHO cells [12,13,14,15]. Such works, performed under steady-state conditions, can provide a snapshot image of intracellular flux distribution and are helpful to analyze and compare specific culture phases. The active fluxes during growth and non-growth phases have been identified [16] as well as during the production phase [17]. Moreover, MFA and FBA approaches are particularly useful to elucidate a metabolic network structure, such as the lactate and glutamine metabolisms, by the use of labelled substrates [15,16,18,19]. However, these MFA and FBA approaches are not predictive neither they can explain metabolic shifts or time-course of a culture behaviour; dynamic approaches being more appropriate for developing an *in silico* platform [20,21,22].

Provost and Bastin [23] and Gao et al. [24] have proposed metabolic models for mammalian cells, linking extracellular fluxes to extracellular concentrations and intracellular fluxes. The model proposed by Provost and Bastin separated the CHO cell culture in growth, transition and death phases, while the model proposed by Gao et al. was able to simulate the exponential and post-exponential phases by dividing the culture in two distinct phases. A similar model by Naderi et al. [25], accounting for dead cells, was able to simulate the decline phase in addition to the growth and plateau phases for several batch and fed-batch cultures after calibration on a batch culture. This kind of models, however, only allows limited predictive capacity since the culture still has to be divided into different growth phases to cope with changes occurring with intracellular fluxes distribution. Nolan and Lee [26] have proposed a dynamic model to simulate external and cytosolic flux kinetics, based on extracellular concentrations and redox state for both batch and fed-batch CHO cell cultures. However, by making the pseudo steady-state assumption on intracellular fluxes, this kind of model can hardly predict intracellular metabolites concentration with time. Based on previous works on plant cells [27,28], a kinetic model describing intracellular and extracellular metabolites concentration, as well as metabolic fluxes variation with time, was transposed to various CHO cell lines [29,30] as well as brain cells [31]. The model successfully described CHO cell batch cultures for recombinant proteins production, such as t-PA [30] and a mAb [29], characterizing the differences between high and low producer clones through model parameters identification and simulations of metabolic flux and flux ratios with culture time. In the present work, the dynamic model was further studied and developed being challenged with different culture media and fed-batch strategies, in other words it was tested against different sets of extracellular conditions. CHO cells have been cultivated under four different experimental conditions, batch and fed-batch cultures using two different culture media, and the metabolomic data sets were used to perform model parameters calibration. Results show that a unique set of model parameter values allows the dynamic metabolic model to describe the different culture conditions tested for the exponential and early plateau phases.

## Materials and Methods

### Cultures

Four different CHO cell cultures were performed to calibrate the model. The CHO-DXB11 cell line, producing a chimeric heavy chain monoclonal antibody (EG2-hFc) [32], has been provided by Dr. Yves Durocher of the NRC (Montreal, Quebec, Canada). Each culture was inoculated at 2x10^5^ cells mL^-1^ in a 2-L Labfors 4 bioreactor (Infors Ag, Bottmingen, Switzerland). Cultures were performed at 37°C and. dissolved oxygen was maintained at 50% of air saturation by surface aeration. pH control at pH 7 was performed by feeding CO_2_ in surface aeration and NaOH (1 M) addition was used when CO_2_ reached a concentration lower than 0.5% of the gas feed. Agitation (pitched-blade impeller) speed was set at 60 RPM at inoculation and was then gradually increased up to 120 RPM to follow the cells oxygen demand, from reaching a maximal O2 concentration of the total gas flow rate of 100%. Cultures were performed until cell viability decreased below 50%. Samples were taken daily for cell counting and cell viability determination, as well as for glucose concentration in the media. In fed-batch cultures, nutrients addition was performed daily to avoid limitations, based on the extracellular glucose concentration, maintaining the concentration above 10 mM. The remaining sample was then centrifuged at 100 *g* and the supernatant was frozen (-80°C) and kept for further analysis of media composition. Cell extractions, for the quantification of intracellular metabolites, were performed as described in Ghorbaniadgdam et al. [29]. Briefly, 3x10^6^ cells were collected and washed 2 times with cold PBS, and then extracted in three steps with cold methanol, while vortexed with 0.2 g of sand (Sigma, Oakville, Canada, cat. # 274739), sonicated in ice bath for 5 min and then centrifuged at 21000 *g* between each steps. Respiration assays were performed daily from 48 h, by closing the bioreactor inlet gas flow rate and decreasing the agitation speed (30 RPM), which allows to minimize the O_2_ transfer while maintaining an homogeneous cell suspension, until the dissolved oxygen level reach 35% of air saturation.

Two different culture media were tested, the Biogro-CHO (Biogro Technologies, Winnipeg, Canada) without glucose and glutamine (see below for concentrations) and the PowerCHO-2 (Sartorius AG, Goettingen, Germany, cat. # WPW-088D) without glutamine (see below for concentration). The Biogro-CHO medium was supplemented with 25 mM and 10 mM glucose (Sigma, Oakville, Canada, cat. # G7021), and with 4 mM and 2.4 mM glutamine (Sigma, Oakville, Canada, cat. # G7513), for batch and fed-batch cultures respectively. The PowerCHO-2 medium was supplemented with 6.5 mM glutamine and 1 mL Anti-Clumping Agent per liter of culture medium (Life Technologies Inc., Burlington, Canada, cat. # 01-0057AE). S3 Table, which allows comparing the different initial conditions for the model, can be used to further compare the known (as determined in this work) composition differences between the two media, especially for the amino acids. The choice of two media was to obtain two different sets of initial conditions to challenge the model behaviour.

Two fed-batch strategies were defined based on the two culture media specific composition. Previous work with the same cell line aiming at identifying limiting nutrients in both Biogro-CHO and PowerCHO-2 media (data not shown) suggested that neither glucose nor amino acids are the substrates limiting cell growth; the individual effects of each compound were evaluated from a fractional experimental plan. Based on those results, with glucose and glutamine being the fastest consumed substrates, medium feed for Biogro-CHO fed-batch cultures was composed of regular medium at 13X glucose (130 mM) and 8X glutamine (25 mM) concentrations (i.e. below solubility limits). The fed-batch culture performed with the PowerCHO-2 medium was fed with a 2.5X concentrated PowerCHO-2 medium (100 mM glucose and 16 mM glutamine). That strategy allowed avoiding early nutritional limitation for the measured components. For both culture media, a fed-batch with daily feeding and a control batch culture were performed in parallel. Samples for all analysis were taken daily, except for the batch culture with the Biogro-CHO, for which the samples were taken every 12 h to better monitor the culture dynamics, since this culture duration showed being shorter than the others.

### Analytical methods

Viable cell concentration was determined by cell counting using a hemocytometer and trypan blue (Sigma, Oakville, Canada, cat # TB154) exclusion method. Extracellular concentrations for glucose, lactate, glutamine and glutamate were analyzed using a YSI 2700 SELECT Biochemistry Analyzer (YSI, Yellow Springs, USA). Ammonia concentration was measured with the Ammonia Assay Kit (Sigma, Oakville, Canada, cat. # A0100). Amino acids concentration were determined using a Agilent 1290 HPLC system (Agilent Technologies, Montreal, Quebec, Canada) as described in Ghorbaniadgdam et al. [29]. Isoleucine and leucine peaks were not distinct, so isoleucine concentration in this work was taken as the sum of isoleucine and leucine concentration, for experimental data as well as in the model. The intracellular extracts were analyzed for nucleotides, organic acids and sugar phosphates concentrations as described in Ghorbaniadgdam et al. [29] using a UPLC MS/MS System (Agilent technologies, Montreal, Quebec, Canada). It was impossible to obtain precise intracellular concentrations for alpha ketoglutarate and NADH for the batch culture using Biogro-CHO medium because these levels were too low to be detected. The same phenomena occurred with pyruvate in the fed-batch culture using the Biogro-CHO medium.

Monoclonal antibody concentration was determined by enzyme-linked immunosorbent assay (ELISA). A Costar 96-well plate (Corning Life Sciences Plastic, Corning, United States, cat. # 3369) was first incubated overnight with Fc specific Anti-Human IgG (Sigma, Oakville, Canada, cat. # I2136) diluted to 1/30000 in phosphate saline buffer (PBS). The wells were washed with PBS solution containing 0.2% Tween 20 (Sigma, Oakville, Canada, cat. #P7949). The remaining sites were then blocked using a solution of PBS containing 0.3 g L^-1^ of Bovine Serum Albumin (BSA) (Sigma, Oakville, Canada, cat. # A7030) incubated for 1 h. The wells were washed and filled with either standard or the sample diluted 1000 to 16000 times in a PBS-BSA solution and incubated for 1 h. The wells were then washed and incubated 1 h with peroxidase conjugated Anti-Human IgG (Sigma, Oakville, Canada, cat. # A0170). The reaction was revealed with 3,3',5,5'-Tetramethylbenzidine (Sigma, Oakville, Canada, cat. # A0170), stopped after 20 min using 2 M H_2_SO_4_ and the absorbance measured at 450 nm with a Victor 3V multilabel reader (PerkinElmer, Waltham, United States).

### Model structure

**Fig 1:**
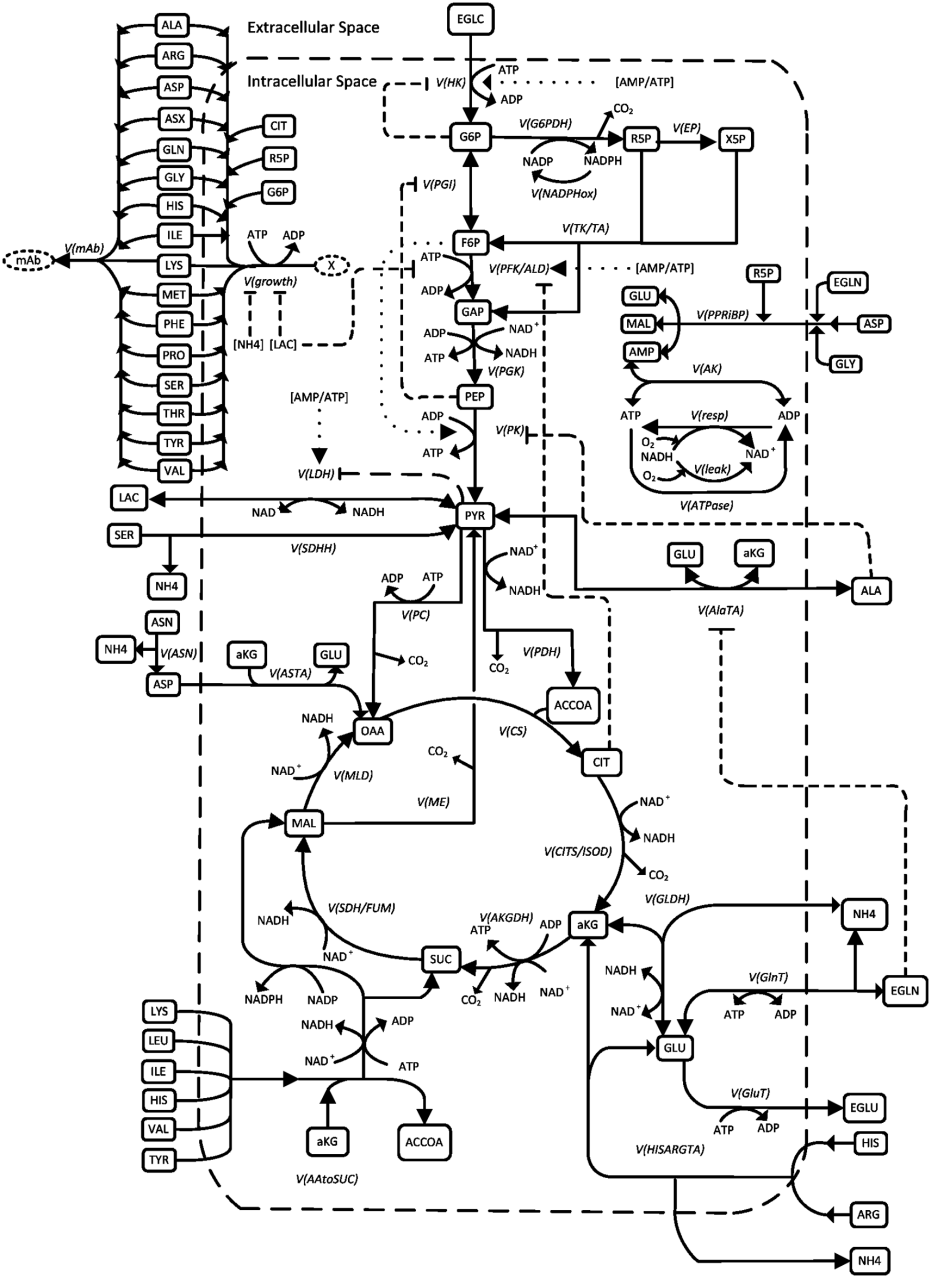
Metabolic network described by the model. Solid lines represent the biochemical reactions, dashed lines the inhibition mechanisms and dotted lines the activation mechanisms.

The mathematical model presented in this work is based on a previous model developed for other two CHO cell lines [29,30,33], as mentioned above. The metabolic network defining the model structure (Fig 1) and the biochemical reactions stoichiometry (S1 Table) have been adapted to the cell line cultured in this study as well as the recombinant mAb produced. The model includes the major pathways such as glycolysis, TCA cycle and pentose phosphate pathway, as well as the pathways for energy production (oxidative phosphorylation) and consumption (ATPases, and anabolic reactions for cell division and mAb synthesis). The major pathways for amino acids metabolism are also described, with glutaminolysis, aspartate and alanine transaminase (*V*_*ASTA*_ and *V*_*AlaTA*_), serine conversion to pyruvate (*V*_*SDHH*_), as well as amino acids conversion to alpha ketoglutarate and succinate in distinct reactions (*V*_*AAtoSUC*_ and *V*_*HISARGTA*_ respectively). The biochemical reactions stoichiometry was preserved from the previous model. Metabolite pool consumption for cell growth was that proposed in Sheik et al. [34] for cell composition, as well as of an average molecular weight of 107.5 g mol^-1^ for the cell proteins in biomass and of a conversion factor of 3.15*10^−4^ gDW 10^−6^ cells. For simplification purposes, the assumption was made that all the lipids within the biomass were derived from citrate, and the nucleotides precursors were issued from ribulose-5-phosphate and glycogen from glucose-6-phosphate. Amino acids consumption for mAb synthesis was calculated from the average IgG1 sequence proposed in Quek et al. [35]. ATP consumption for both cell growth and mAb synthesis are as described in Nolan & Lee [26]. Also, a subset of the non-measured metabolites, and associated enzymes, that were considered in the previous model (creatine, phosphocreatine, coenzyme-A, phosphate and intracellular oxygen) were not considered here.

### Mathematical description of the metabolic fluxes

Most of fluxes kinetics are as previously described [29,30,33] (S2 Table). All kinetic equations in this work are based on mechanistic formulations known as biologically relevant, rather than using empirical equations. Reactions rate are composed of multiplicative Michaelis-Menten kinetics accounting for each substrates involved. In the case of energetic nucleotides, ratios are used (AMP/ATP, ATP/ADP, NADH/NAD, NADP/NADPH, ADP/ATP, NAD/NADH, NADPH/NADP). This approach was successful in another work on the modeling of the metabolism of skeletal muscle cells [36] as well as in our previous work on a similar modelling approach applied to another CHO cell line [29, 30, 33]. The regulation of glycolysis is as previously described [33], with feedback inhibition phenomena of hexokinase (*V*_*HK*_), phosphoglucose isomerase (*V*_*PGI*_) and reverse lactate dehydrogenase (*V*_*rLDH*_), by glucose-6-phosphate, phosphoenolpyruvate and pyruvate, respectively. The AMP-to-ATP ratio activates phosphofructokinase (*V*_*PFK*_), lactate dehydrogenase (*V*_*LDH*_) and *V*_*HK*_, while fructose-6-phosphate activates pyruvate kinase (*V*_*PK*_). The reactions involving an inhibition mechanism were described according to the mathematical formulation of a non-competitive inhibition (Eq 1). However, the reaction mechanism used to describe activation phenomena is that for non-essential activation as proposed in [37] (Eq 2).

$$V=\frac{V_{\text{max}}[S]}{\left[S]+K_{m}(1+[I]/K_{I}\right)}$$ (1)

$$V=\frac{V_{\text{max}}[S](1+\beta [\text{A}]/\alpha K_{A})}{K_{S}(1+[A]/K_{A})+[S](1+[A]/\alpha K_{A})}$$ (2)

In the present work, *V*_*PFK*_ is inhibited by intracellular citrate and extracellular lactate [38,39], and *V*_*PK*_ is inhibited by alanine [40,41]. The reverse reaction of alanine aminotransferase (*V*_*rAlaTA*_) is also inhibited by glutamine to account for the switch from alanine production to alanine consumption when glutamine level is low, as observed in our experimental data. Reverse reactions are also modeled for glutamine synthetase (*V*_*GlnT*_), glutamate dehydrogenase (*V*_*GLDH*_), lactate dehydrogenase (*V*_*LDH*_), glutamate transport (*V*_*GluT*_) and adenylate kinase (*V*_*AK*_), but not for *V*_*ASTA*_ because there was no evidence from our measurements of a net production of extracellular aspartate, but only a net consumption.

The cell specific growth rate is modeled as a multiplicative Michaelis-Menten mechanism accounting for major precursors of cell building blocks, an approach that has been previously successfully applied to plant [27] and mammalian cells [29,30,31]. All extracellular amino acids included in the model were considered, as well as the intracellular levels of glucose-6-phosphate, citrate and ribulose-5-phosphate, which act as the respective precursors for glycogen, proteins and lipids, and nucleotides such as DNA and RNA. For each of those species, a different affinity constant was determined, to represent the situation when one species, for instance glutamine, is nearly depleted without significantly affecting the growth rate. A specific set of affinity constants was also used to describe the mAb production rate. Finally, inhibitory effects of lactate and ammonia on the growth rate were added to account for their accumulation in the culture media. A non-competitive inhibition mechanism was applied [42], with a distinct affinity constant for lactate regarding the growth (Equation 33 in S2 Table) and *V*_*PFK*_ (Equation 3 in S2 Table) reactions.

### Sensitivity analysis

The model structure and flux kinetics formulation resulted in a large number of parameters (139). Before proceeding with the calibration step of the model, the parameters were analysed in order to identify and only optimize the value of the most sensitive ones. The Morris Screening method was used for determining the ranking of the global sensitivity of each parameters, an approach previously applied to a similar dynamic model [43,44]. This technique allows to cover efficiently a wide sample space returning the distribution of the elementary effect in that space [45]. The absolute value of the mean indicates the sensitivity of the parameter and the standard deviation gives an approximation of the linearity of the parameter. The elementary effect of a parameter *p*_*i*_ on the output of an objective function *f* can be defined as follow (Eq 3):

$${\text{EE}}_{\text{i}}(\text{p})=\text{f}({\text{p}}_{1},{\text{p}}_{2},\ldots ,p_{\text{i}-1},{\text{p}}_{\text{i}}+\Delta ,{\text{p}}_{\text{i}+1},\ldots ,{\text{p}}_{\text{k}})/\Delta$$ (3)

**Fig 2:**
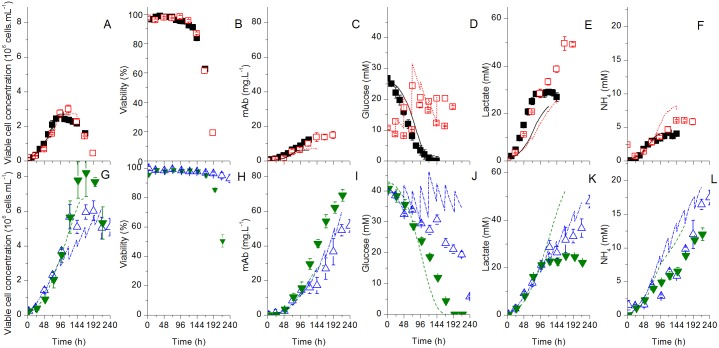
Experimental and model simulation results for cells and major extracellular components. Experimental (symbols) and model simulation (lines) results for the batch (filled symbols) and the fed-batch (empty symbols) cultures using the Biogro-CHO medium (squares) or the PowerCHO-2 medium (triangles). Model simulated results for the batch cultures using Biogro-CHO (straight line—black) and PowerCHO-2 (dashed lines—green), and fed-batch cultures using Biogro-CHO (doted line—red) and PowerCHO-2 (dashed and dotted lines—blue) for all results except for cell viability in B. All simulations obtained from a unique model. A-F are for the Biogro-CHO medium, G-L for the PowerCHO-2 medium.

**Fig 3:**
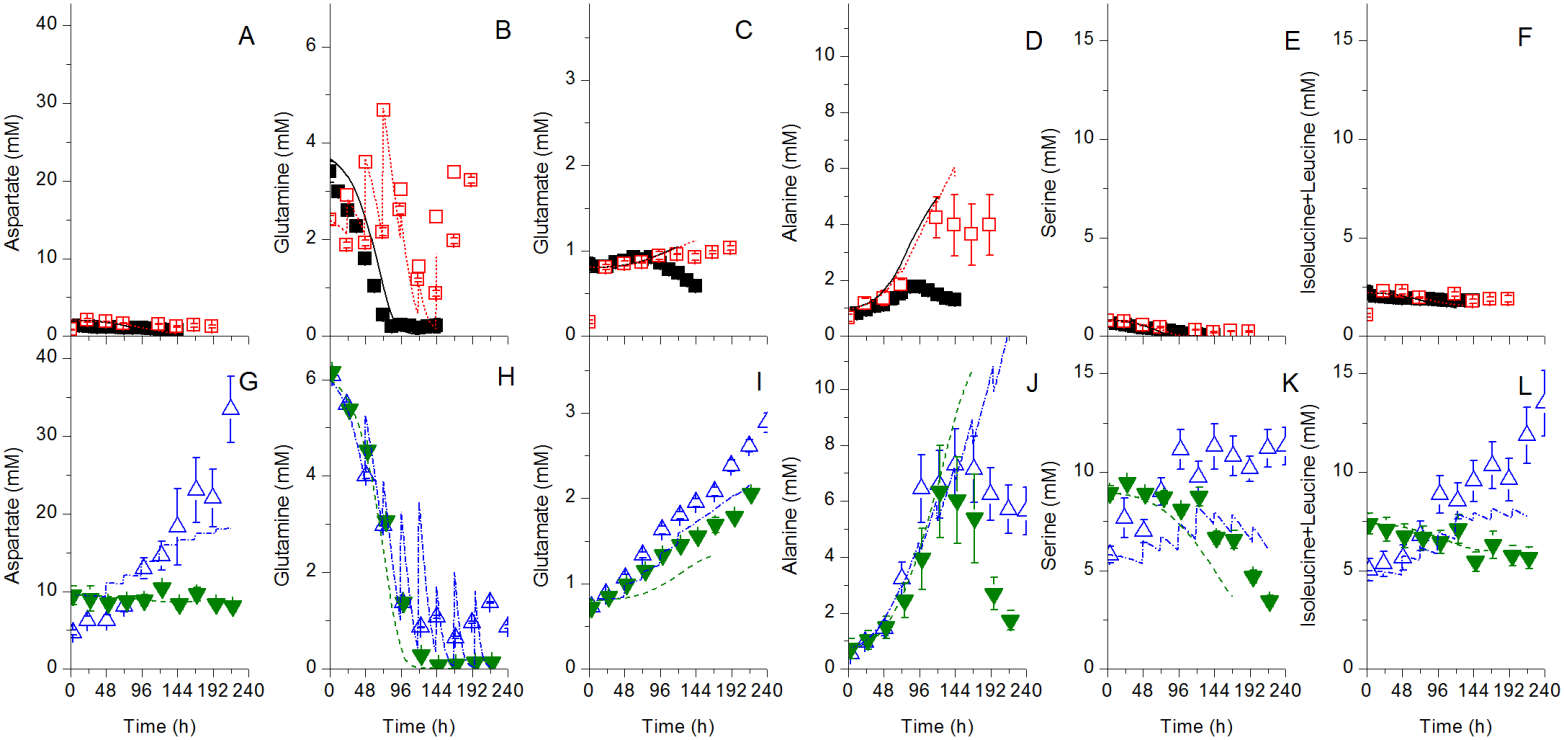
Experimental and model simulation results for major extracellular amino acids. Same conditions and symbols than in Fig 2 applied. A-F are for the Biogro-CHO medium, G-L for the PowerCHO-2 medium.

**Fig 4:**
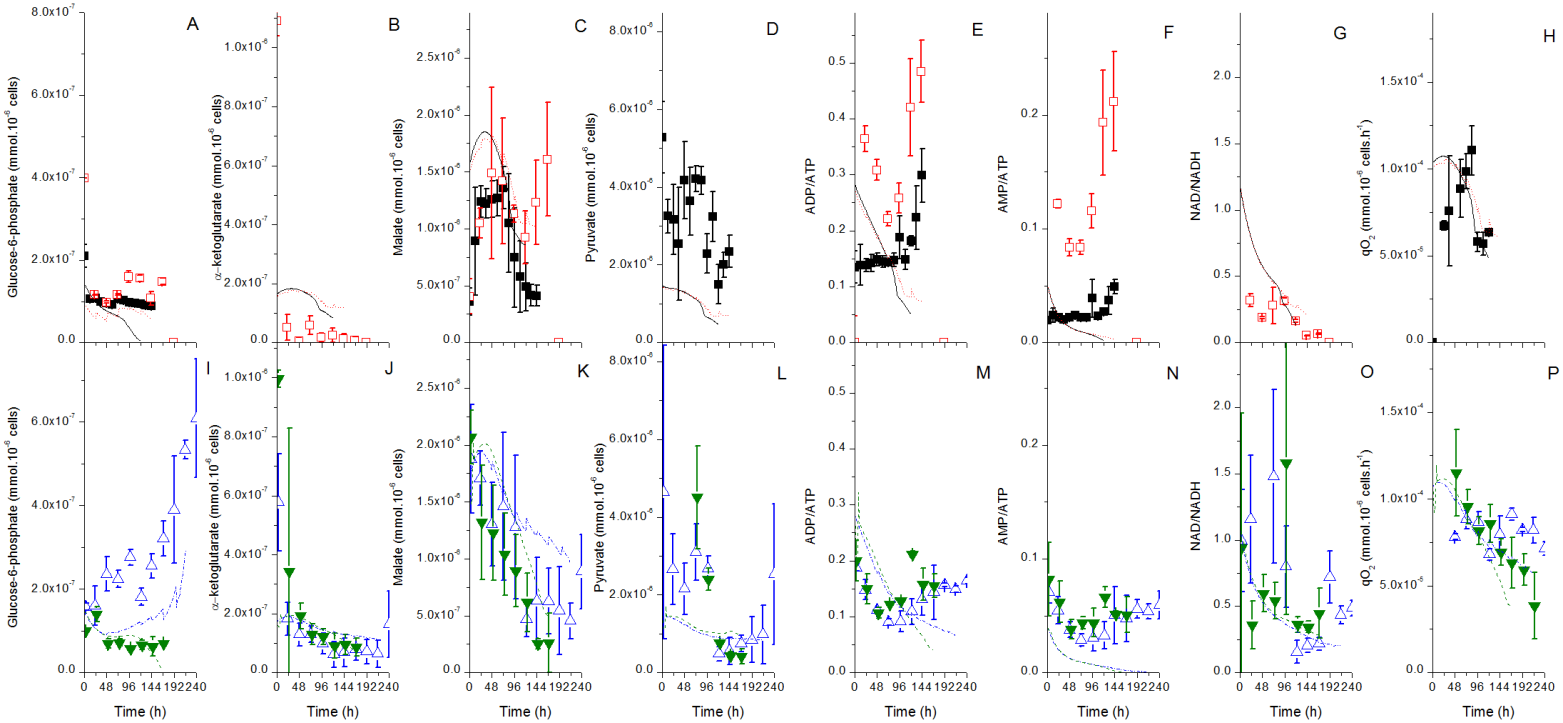
Experimental and model simulation results for major intracellular components. Same conditions and symbols than in Fig 2 applied. A-H are for the Biogro-CHO medium, I-P for the PowerCHO-2 medium.

The elementary effect was calculated 35 times for each parameter using a step Δ of 2/3 and the boundaries were set to ±25% of the initial guess. The outputs were the function evaluation at the experimental sampling times for the selected 32 outputs chosen for optimization (the ones represented in Figs 2–4 and S1–S3 Figs). The outputs were weighted using their standard deviation, as it is the case in the parameter optimization routine. The mean of all the outputs in absolute value at all the time points was used to determine a single sensitivity value for each parameter. The parameters were then ranked according to their global sensitivity and the most sensitive ones were identified. The use of the mean for all outputs was to allow the result to be a single value that can be easily ranked, considering the large number of outputs involved.

### Parameter estimation and confidence intervals

The initial guess for all parameters were those from previous work [29]. The *K*_*m*_’s of the specific growth rate and the mAb production rate were first assumed to be lower than the *K*_*m*_ *’s* of the biochemical reactions. The first step consisted in manually changing the parameter values verifying the reliability of simulations compared to experimental data, for the complete culture duration. Then, the sensibility analysis was performed to identify the most sensitive parameters. Finally, using the least square optimization function from MATLAB ToolBox (MathWorks), the optimization step was performed with the following objective function, the parameter vector *p* representing the 20 most sensitive parameters:

$$\text{min}(\Sigma_{\text{n = 1}}^{\text{N}}\Sigma_{\text{t = 1}}^{\text{T}}{\left(\frac{{\text{y}}_{\text{n,t}}^{\text{exp}}-{\text{y}}_{\text{n,t}}(\text{p})}{\sigma_{\text{n,t}}}\right)}^{2})$$ (4)

where y_n,t_(p) is the *n* ^th^ model output evaluated at the *t* ^th^ experimental time, y^exp^ is the experimental data and σ is the standard deviation of the experimental measurement. Those three steps were repeated in the same order until optimisation results yielded realistic (biologically) parameter values with sufficiently narrow confidence intervals and minimal residual error. At each iteration, the initial guesses were changed and the parameters to be optimized were selected according to the sensibility analysis results. The results presented in this work correspond to the final step of this process, which correspond to the minimal residual error attained.

Experimental data from 24 h after inoculation to when the cell viability decreased under 95% were considered, and the molecular species used for optimization are the same as for sensitivity analysis. The parameter values were also normalized to their specific order of magnitude (10^n^) to improve the optimization process by allowing similar weight to each parameter. The lower bounds for all parameters were set to 0. Finally, flux values and the 95% confidence interval for each optimized parameter were calculated using the MATLAB built-in functions *nlparci* and *nlpredci* as well as the Jacobian matrix from the *lsqcurvefit* function. When available, initial conditions were taken from experimental data or from [29] otherwise (see S3 Table for initial conditions).

Five different optimization procedures were performed: using the four culture data sets, focusing on the two cultures performed with Biogro-CHO, on the two cultures performed with PowerCHO-2, using data from the two fed-batch cultures or using data from only the two control batch cultures. The parameter values were initially obtained considering all four cultures, and those values were taken as the initial guesses feeding the four other optimizations. The goal was to be able to compare the parameter confidence intervals from two different subsets of the whole data sets. First, the batch cultures as a whole were compared to the fed-batch culture, then the cultures with Biogro-CHO medium, independently of their culture mode, were compared to the PowerCHO-2 cultures. In a similar way, data sets from each culture were also divided into two phases based on cell growth behaviour before and after 72 h, which corresponds approximately to the mid-exponential phase. The parameters were first optimized with a data set that corresponded to the first 72 h for all cultures, followed by a second optimization step from 72 h to the end of the culture, for all 4 cases. The parameter initial guesses (at t = 0) were the same in each case. The goal was to evaluate the appropriateness of dividing the model in 2 culture phases.

## Results and Discussion

### The culture medium is a primary factor in cell culture behaviour

All cultures were performed until cell viability reached 50%, which occurred at 144 h and 216 h for batch cultures using Biogro-CHO and PowerCHO-2 respectively, and after 192 h and 360 h for the fed-batch cultures (Fig 2B and 2H). The PowerCHO-2 medium composition thus allowed maintaining high cell viability for an extended time period in batch and fed-batch cultures compared to Biogro-CHO. Moreover, both PowerCHO-2 cultures led to higher maximum viable cell concentrations (Fig 2A and 2G) and higher mAb titers (Fig 2C and 2I) (6.0x10^6^ cells mL^-1^ and 69 mg mAb L^-1^ for the fed-batch culture, and 8.2x10^6^ cells mL^-1^ and 67 mg mAb L^-1^ for the batch culture) than the cultures using Biogro-CHO (3.0x10^6^ cells mL^-1^ and 14.9 mg L^-1^ for the fed-batch culture, 2.7x10^6^ cells mL^-1^ and 12.1 mg L^-1^ for the batch culture). High non-limiting glucose condition (Fig 2D), such as in the Biogro-CHO fed-batch culture, has not resulted in higher culture performance, nor maintaining high glutamine concentration, since the PowerCHO-2 batch culture exhibits sustained growth in absence of glutamine (Fig 3H). However, the PowerCHO-2 medium contains more aspartate, asparagine, isoleucine, arginine and serine than the Biogro-CHO (Fig 3), most probably fuelling cell metabolism for an extended culture period. The differences in culture behaviour between the two media can thus be attributed to the balance of PowerCHO-2 components concentration and ratios. Therefore, with a significant medium effect on cell behaviour, the selected experimental space appeared appropriate for studying the dynamic model robustness.

### Sensitive parameters are of glycolysis regulation, the energetic metabolism and *V*_*max*_’s

The model sensitivity analysis was performed as described in the Material and Methods section, by first considering all four experimental data sets. The parameters for which the mean value of the elementary effect was significantly high (i.e. lower than 0.6) were identified as the most sensitive and were kept for the parameter optimization subsequent steps. The number of parameters was restrained to the most sensitive ones in order to increase the accuracy and the efficiency of the optimization process.

Of the 20 sensitive parameters, 11 are maximal rate constants (*V*_*max*_) and 4 are affinity constants (*K*_*m*_), and the remaining parameters are inhibition (2) and activation constants (3). Growth (*V*_*maxgrowth*_), glutamine metabolism (*V*_*maxGlnT*_), glycolysis (*V*_*maxHK*_, *V*_*fmaxPGI*_, *V*_*maxPGK*_ and *V*_*maxPK*_) and energetic metabolism *(V*_*maxATPase*_, *V*_*maxresp*_, *V*_*maxleak*_, *V*_*rmaxAK*_ *and V*_*fmaxAK*_) were among sensitive parameters. Interestingly, the pathways with sensitive *V*_*max*_ parameters are among the ones previously identified as sensitive but for other CHO cell lines [29,30]. Of the 4 sensitive *K*_*m*_, 3 are associated to cell energetics with *K*_*mNADH*_ and *K*_*mATP*_, respectively involved in NADH and ATP consumption, and *K*_*mADP/ATP*_, which affects seven energy-producing reactions. Within the regulation constants, the sensitivity of *K*_*mG6P*_ is explained by the fact that glucose-6-phosphate level directly controls the glycolytic influx from a retro-inhibition term, with *K*_*dG6P*_ also being a sensitive constant. The value of *K*_*mG6P*_ has a direct impact on the glucose-6-phosphate level, which in return affects greatly the glycolytic flux. Finally, sensitivity of *K*_*dNH4growth*_ may be linked to high NH_4_ concentration accumulating in the media and thus expected to limit cell growth. The fact that 3 of those sensitive regulation constants, along with the *V*_*rmaxAK*_ *and V*_*fmaxAK*_, are closely linked to the AMP-to-ATP ratio highlights the crucial role of this regulation pathway on glycolysis. Moreover, the AMP-to-ATP ratio is an efficient regulator of cell metabolism, since it varies faster than the ADP-to-ATP ratio [46] and the fact that it affects several glycolytic enzymes, explaining the sensitivity of the parameters that are linked to this ratio.

Although only *V*_*max*_ constants have been identified as sensitive in our previous work with CHO cells [29,30], Nolan & Lee [26] did also identified as sensitive some regulatory constants from the glycolysis, especially in regards to lactate profile, studying a dynamic model for another CHO cell line. Finally, performing a sensitivity analysis on a dynamic model for plant cells [47], Mailier et al. [44] identified the concentrations of phosphate, nitrate and sulphur, which acted as regulators, among the most sensitive parameters.

### A single set of parameters allows describing cell behaviour for various culture conditions

**Fig 5:**
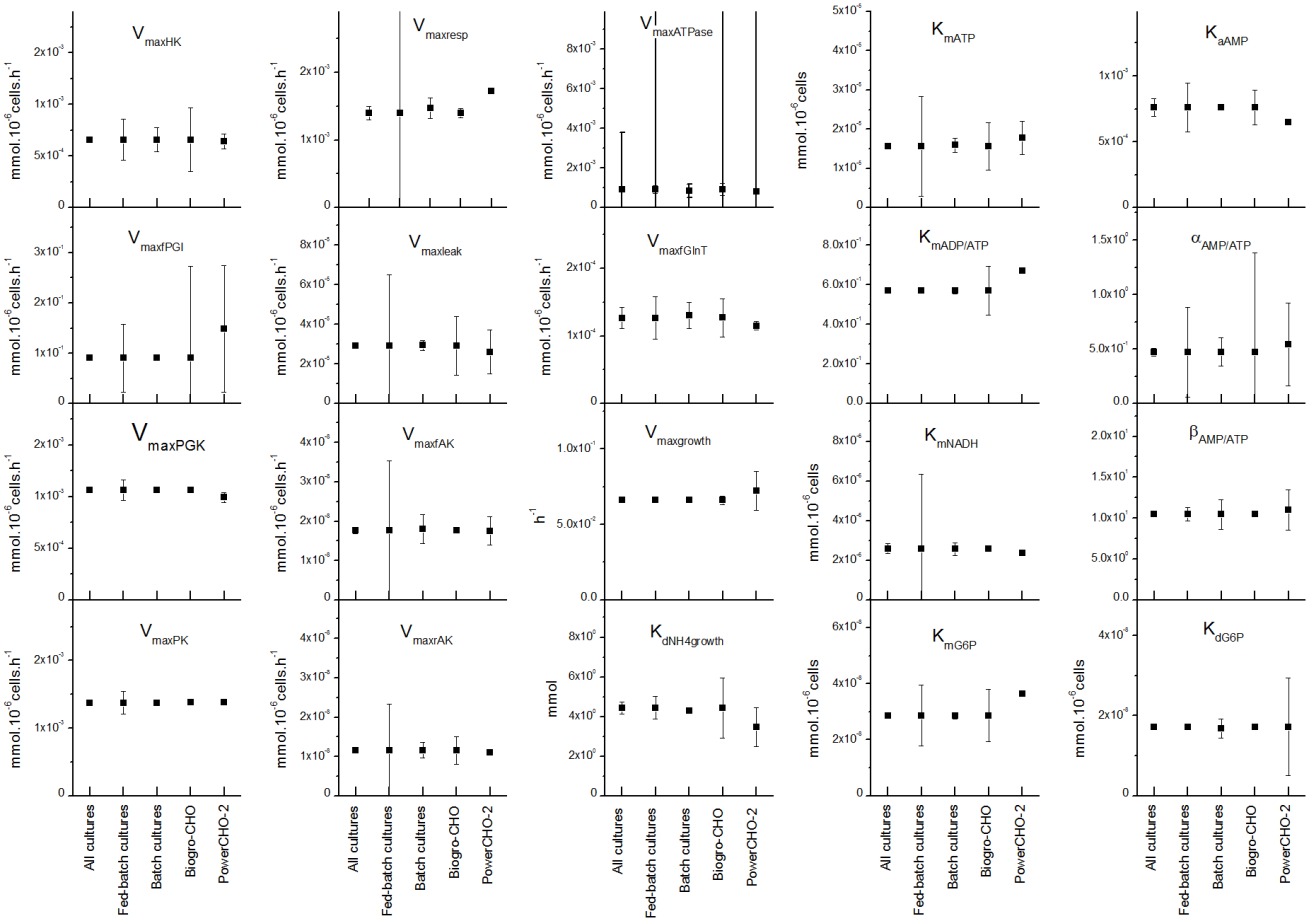
Parameter estimates with confidence intervals for different media and culture modes. The parameter values and their confidence region are compared when using for the optimization: All data from all cultures, Data from fed-batch cultures, Data from batch cultures, Data from cultures using Biogro-CHO and Data from cultures using PowerCHO-2.

The model was first calibrated using all four sets of the experimental data obtained in this work, as described in the Material and Methods section. The resulting model was then used to produce simulations of experimental data (Figs 2–4 and S1–S3 Figs). It was then evaluated if model parameters are culture medium and/or culture mode dependent. Therefore, independent model calibrations were performed on all possible combinations of experimental data subsets. Model parameters were then compared for their confidence regions (Fig 5 presents the differences between the culture mode and the culture medium, see S5 Table for numerical values with 95% confidence intervals). There are only few parameters that are significantly differing according to the data sets used for optimization. In fact, there are no differences between the batch and the fed-batch results, while there are only 3 parameters out of 20 being modified comparing the two culture media. *V*_*maxPGK*_ is slightly (but significantly) lower in PowerCHO-2, while lower *K*_*mNADH*_ and higher *V*_*maxresp*_ for the PowerCHO-2 could reveal a higher respiration rate for this medium. The absence of difference between batch and fed-batch cultures is in agreement with another dynamic model describing hybridoma metabolism [48]. In that case, among half-saturation constants for all metabolic reaction rates, only the asparagine half-saturation constant for asparagine consumption differed simulating batch and fed-batch culture data, while 9 maximum reaction rates on 11 were identical, with only the *Vmax* for the conversion of glutamate to proline and for the conversion of asparagine to asparatate being different. The overall performance of the model in the present work suggests that a unique model can be used to adequately simulate different fed-batch strategies on different culture media with a unique set of parameter values, which was not the case when analysing different mAb recombinant CHO clones [29]. Moreover, the calibration of the model parameters on each experimental data set taken separately negatively impacts on the magnitude of the confidence intervals (Fig 5), thus reducing the overall model precision. The choice of a model with a single set of parameters thus seems more interesting since, the parameters are more accurate when using a wide array of experimental data obtained from different experimental spaces, and it allows the model to be used in a more robust way. Given that normally each medium is developed specifically for a cell line and that its composition is highly complex, with for instance tedious to measure growth factors, it should be assumed that a dynamic metabolic model is expected being specific to each medium to perform well. However, in our case a single model showed being efficient at simulating the major metabolic differences between two different media applied to the same cell line. Indeed, it thus seems from this work and previous observations [29] that a model has to be cell line specific but it can be a useful tool to simulate cell behaviour testing various culture media and culture conditions.

### A single set of parameters allows describing exponential and plateau growth phases

**Fig 6:**
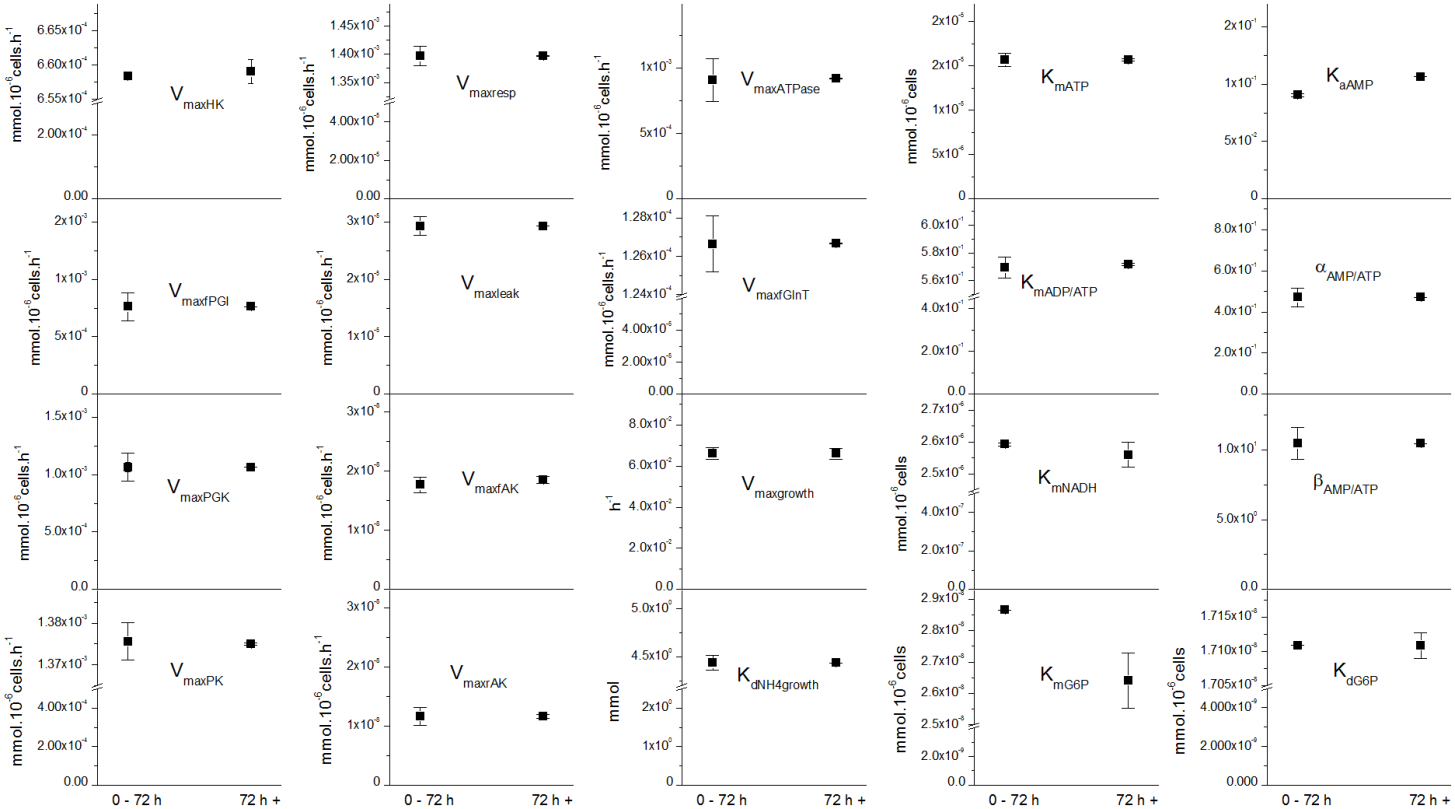
Parameter estimates with confidence intervals for different growth phases. Parameter value (black square) and confidence region when using data before the mid-exponential growth phase at 72 h or after the mid-exponential growth phase for optimization.

Moving further challenging the model structure performance, we have evaluated the benefit of dividing the model to describe two distinct but successive phases: before (< 72 h) and after (> 72 h) mid-exponential growth phase. This time point approximates the moment where growth and other major fluxes start declining. Although a fully dynamic model that includes pathway regulation, such as in this work, is thought being able to cope with cell behaviour variation with time, the aim was to evaluate the effect on model simulations accuracy. The idea of dividing the culture into two [16,24,49], three [50] or four [17] distinct culture phases has also been proposed to model mammalian cells behaviour. In these works, indeed, metabolic flux analysis (MFA) studies have revealed significant differences of flux levels and distribution at different stages of a culture, under quasi-steady state condition however. In the present work, the confidence regions overlap (confidence intervals (95%) in Fig 6, and numerical values in S6 Table) for all except for two parameters comparing before and after 72 h. The estimated value of *K*_*mG6P*_ is slightly lower after 72 h (2.6*10^−8^ mmol.10^-6^cells versus 2.9*10^−8^ mmol.10^-6^cells), which could indicate the limitation of glucose-6-phosphate depletion, and the value of *K*_*aAMP*_ is higher (0.11 versus 0.09), which may reveal a decrease of glycolytic activity and lactate production at the mid-exponential phase. Overall, although simulation error can be lower when changing the value of these two parameters, the structure of the model also shows that it is possible to describe the whole culture behaviour from inoculation to the decline phase with a unique set of parameter values. While some discrepancies between experimental and simulated data for some species can still be observed, this model is, to the best of our knowledge, the only capable to simulate intracellular and extracellular concentrations for batch and fed-batch cultures. It thus represents a first step in the development of a predictable *in-silico* platform of cell behaviour. Moreover, since the model does not describe cell viability, and considering that the metabolomic data remain reliable at high cell viability, it was decided to limit the further evaluation of the model to the data sets with cell viability level of 95% and above. The decline phase is thus not described nor simulated, limiting the scope of the model to the exponential and early plateau phase.

Therefore, a unique model structure with a unique set of parameter values has been used to simulate each culture (Figs 2–4). The simulations are in general within the experimental data ± error range, which corresponds to the standard deviation. The model simulates adequately the cell (Fig 2A and 2G) and mAb (Fig 2C and 2I) concentrations with time for the two media. Glucose (Fig 2D and 2J) and glutamine (Fig 3B and 3H) profiles match the experimental data for batch cultures, while the model somewhat underestimates glucose consumption for the fed-batch culture using PowerCHO-2 but overestimates glutamine consumption for both fed-batch cultures. It should be reminded that the only model inputs are the parameter values, the initial conditions and the feed volume and composition. Since glucose and glutamine are fed in greater quantities, the deviations could be explained by the cumulative error at each feed.

The model also simulates cell respiration rate (Fig 4H and 4P) and related TCA metabolites such as malate (Fig 4C and 4K) and NAD/NADH ratio (Fig 4G and 4O). The accumulation of glucose-6-phosphate in the fed-batch culture using PowerCHO-2 is also accurately simulated (Fig 4I). The AMP-to-ATP ratio is somehow underestimated by the model (Fig 4F and 4N) but, however, it follows the general trend of experimental data except for the fed-batch culture using the Biogro-CHO medium. The metabolic shift from alanine production to alanine consumption (Fig 3D and 3J) is not as pronounced in the simulations compared to experimental data. The initial underestimation of pyruvate concentration (Fig 4L) might be related to the overestimation of the direct *V*_*AlaTA*_ flux in the latter stages of the culture, since the pyruvate concentration stays constant in the simulations compared to experimental data. Also, in the model, the *V*_*AlaTA*_ reverse reaction is inhibited by extracellular glutamine, while the shift seems closely related to glutamine depletion, suggesting that the chosen descriptive mechanism might not be optimal. Moreover, the uptake rate for some amino acids seems to be overestimated (e.g. tyrosine on S2A and S2F Fig and valine on S2B and S2G Fig) or underestimated (e.g. asparagine, S2E and S2J Fig) for some others. A strategy to enhance the simulation fit for these compounds would consist in optimizing cell and mAb composition (i.e. stoechiometry); understanding that the cell and mAb compositions can differ from the values proposed in literature [34,35]. Moreover, this could also contribute improving simulation of the pentose phosphate pathway components (xylose-5-phosphate shown in S3D and S3H Fig), which are currently overestimated by the model. Since ribulose-5-phosphate is consumed for biomass synthesis, this pathway is highly sensible to growth rate and thus to the cell content in nucleotide precursors.

### Fed-batch culture mode enables maintaining cell metabolic activity

**Fig 7:**
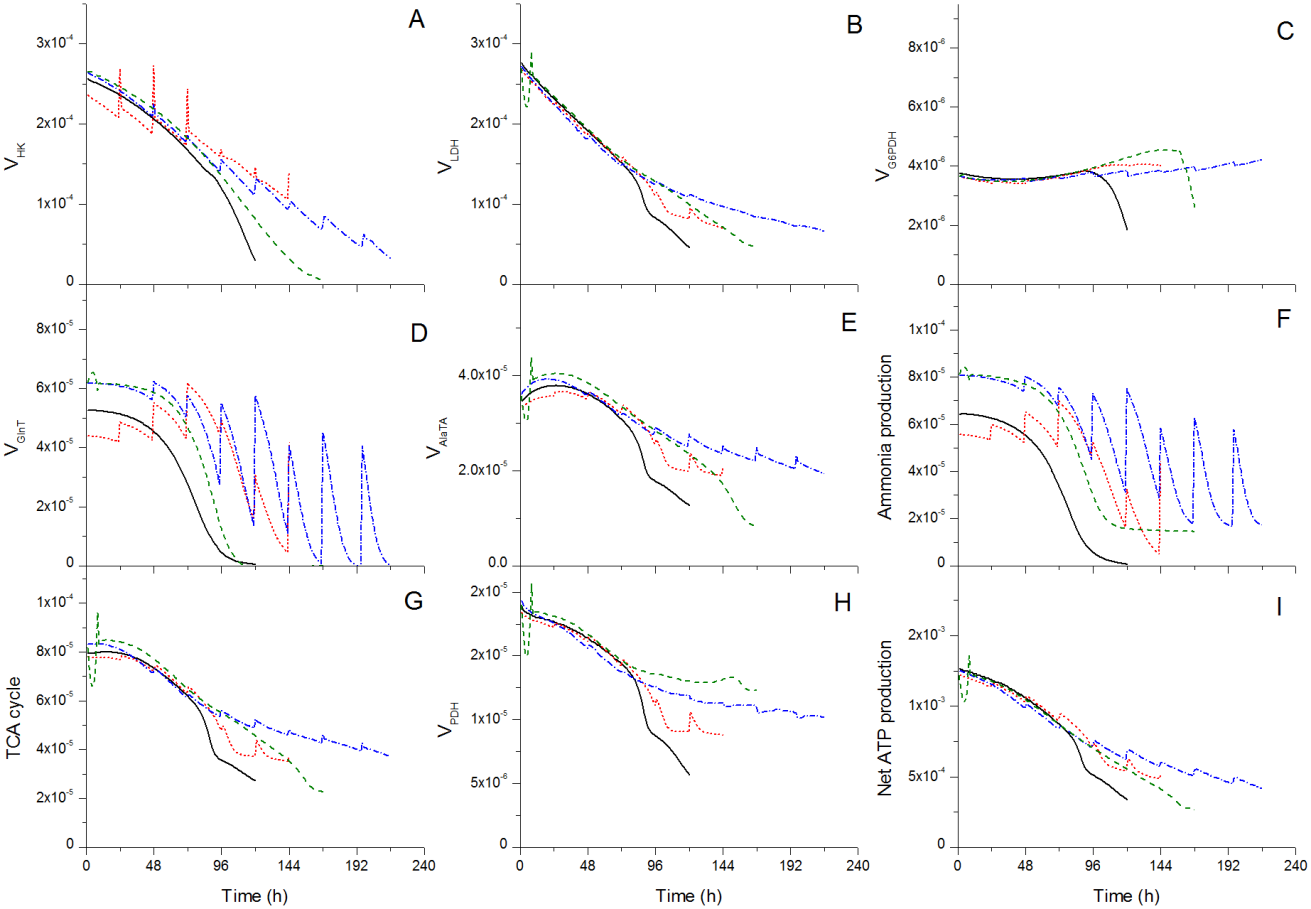
Model simulation of metabolic fluxes. Metabolic fluxes (all in mmol.10^-6^cells.h^-1^) were simulated for the same conditions as in Figs 2–4 for batch cultures using Biogro-CHO (straight line—black) and PowerCHO-2 (dashed line—green), and fed-batch cultures using Biogro-CHO (dotted line—red) and PowerCHO-2 (dashed and dotted lines—blue). Ammonia production (Fig 7F) is defined as *V*_*GLDH*_+*V*_*GlnT*_+*V*_*SDHH*_+*V*_*ASN*_+*V*_*HISARGTA*,_ and TCA cycle flux (Fig 7G) is defined as the sum of the reactions entering in (*V*_*GLDH*_+*V*_*PDH*_+*V*_*AlaTA*_+*V*_*PC*_+5*V_*AAtoSUC*_), and finally the net ATP production flux (Fig 7I) consists in the sum of the ATP-producing metabolic fluxes (*V*_*PGK*_ + *V*_*PK*_ + *V*_*AKGDH*_ + *V*_*PC*_ + *V*_*GlnT*_ + 2*2.5*V_*resp*_).

The model, which showed being efficient at describing cell behaviour for the four culture conditions assessed, was then used as an *in silico* platform to perform a dynamic MFA study extracting tedious to impossible to obtain data from the same cultures. Major fluxes (Fig 7) at all time points for all four cultures, as well as the numerical results with 95% confidence intervals at 48 h (mid-exponential growth phase) and 96 h (plateau growth phase) (S7 and S8 Tables) are shown. Simulated metabolic fluxes are in agreement with literature on CHO cells. Glucose consumption rate (*V*_*HK*_) was similar to that for lactate consumption (*V*_*LDH*_) rate (~ 2x10^-4^ mmol.10^-6^cells.h^-1^ after 48 h), a value range that is consistent with literature with [1–2]x10^-4^ mmol glucose.10^-6^ cells.h^-1^ and [1–3]x10^-4^ mmol lactate.10^-6^ cells.h^-1^ at exponential phase [16,17,50]. The pyruvate dehydrogenase flux, which connects glycolysis and TCA cycle shows a low value range of 1.6 ±0.2 to 1.7±0.2 10^−5^ mmol.10^-6^cells.h^-1^ that is close to a value of 2.71x10^-5^ reported by Templeton et al. [17], while an higher value (13.5x10^-5^ mmol.10^-6^cells.h^-1^) has been reported by Ahn & Antoniewicz [16] at exponential phase. The pentose phosphate pathway flux value (3.4±0.3–3.7±0.3 10^-6^mmol.10^-6^cells.h^-1^ at 48 h) is similar to values reported at exponential phase with 1.8±0.2 mmol.10^-6^cells.h^-1^ [16] and at early exponential phase 1.25x10^-6^ cells.h^-1^ [17].

There are very few differences in the flux levels for the first 72 h of the culture when the four cases are compared. At 48 h, the glutamine consumption rate (*V*_*GlnT*_) is significantly lower for the batch culture using Biogro-CHO than for the fed-batch culture using PowerCHO-2. Ammonia production rate (*V*_*GLDH*_+*V*_*GlnT*_+*V*_*SDHH*_+*V*_*ASN*_+*V*_*HISARGTA*_) seems lower for Biogro-CHO compared to PowerCHO-2, although the difference is only significant when comparing the batch mode with that medium to other cultures. This result was expected since Biogro-CHO medium contains significantly less glutamine at inoculation (and after 48 h), thus limiting *V*_*GlnT*_ and indirectly ammonia production.

Although all metabolic fluxes show a decreasing trend whatever the culture conditions from 48–96 h, significant differences of behaviour raised in the latter culture stages comparing flux levels among cultures. This flux decrease was noticeable for glucose consumption (*V*_*HK*_, Fig 7A), lactate production (*V*_*LDH*_, Fig 7B), glutamine consumption (*V*_*GlnT*_, Fig 7D), alanine production (*V*_*AlaTA*_, Fig 7E), the global TCA cycle activity (Fig 7G, defined as the sum of the fluxes that enter the TCA cycle or *V*_*GLDH*_+*V*_*PDH*_+*V*_*AlaTA*_+*V*_*PC*_+5*V_*AAtoSUC*_), and for the net ATP production rate (Fig 7I, defined as the sum of all ATP-producing fluxes or *V*_*PGK*_ + *V*_*PK*_ + *V*_*AKGDH*_ + *V*_*PC*_ + *V*_*GlnT*_ + 2*2.5*V_*resp*_). In the case of *V*_*HK*_ and *V*_*LDH*_, the only significant difference after 96 h is observed between the batch culture using Biogro-CHO and the other cultures, a result that may be due to faster glucose depletion in the former case. However, both the glutamine consumption rate and the ammonia production rate show maintaining their activity levels in the two fed-batch cultures. This result indicates that the feed strategy was able to maintain glutamine metabolism towards the TCA cycle and to limit glutamine depletion. Unlike glutaminolysis, glycolysis is not maintained at the same activity level in fed-batch cultures even if glucose was not limiting. This could be due to the inhibition effect of lactate and alanine on phosphofructokinase (*V*_*PFK*_, equation 3 in S2 Table) and pyruvate kinase (*V*_*PK*_, equation 5 in S2 Table) respectively. The value of the inhibition term of lactate on glycolysis under fed-batch conditions decreased from 0.89 initially to 0.29 after 96 h, while the effect of alanine is much smaller (0.96 to 0.86), thus decreasing phosphofructokinase less than lactate. Lactate accumulation is thus thought causing a significant inhibition phenomenon in fed-batch. The dynamic structure of the model as well as the inclusion of the main mechanisms involved in glycolysis regulations result in a robust but reactive biosystem; the decreasing of a single flux can affect all the other glycolytic fluxes, including glucose consumption (*V*_*HK*_) and lactate production rates. The pentose phosphate pathway global activity (*V*_*G6PDH*_, Fig 7C), however, remains constant throughout the culture unlike glycolysis to which it is intimately related. Interestingly, the pyruvate dehydrogenase activity level (*V*_*PDH*_, Fig 7G) shows being linked to the medium composition rather than to the culture mode. Indeed, the values in Biogro-CHO after 96 h (0.75±0.13 10^−5^ mmol.10^-6^cells.h^-1^ for batch culture, 0.75±0.19 10^−5^ mmol.10^-6^cells.h-^1^ for the fed-batch culture) are significantly lower than the ones for PowerCHO-2 (1.36±0.17 10^−5^ mmol.10^-6^cells.h-^1^ for batch culture, 1.26±0.15 10^−5^ mmol.10^-6^cells.h-^1^ for the fed-batch culture). Since *V*_*PDH*_ activity relies on the intracellular concentration of pyruvate and on the NAD/NADH ratio in our model, it may be affected indirectly by the culture medium composition in the latter culture stages from extracellular metabolites concentration. However, further investigations are required to clearly elucidate the cause behind this difference of behaviour.

### Fed-batch culture condition enhances the stability of metabolic flux distribution

**Fig 8:**
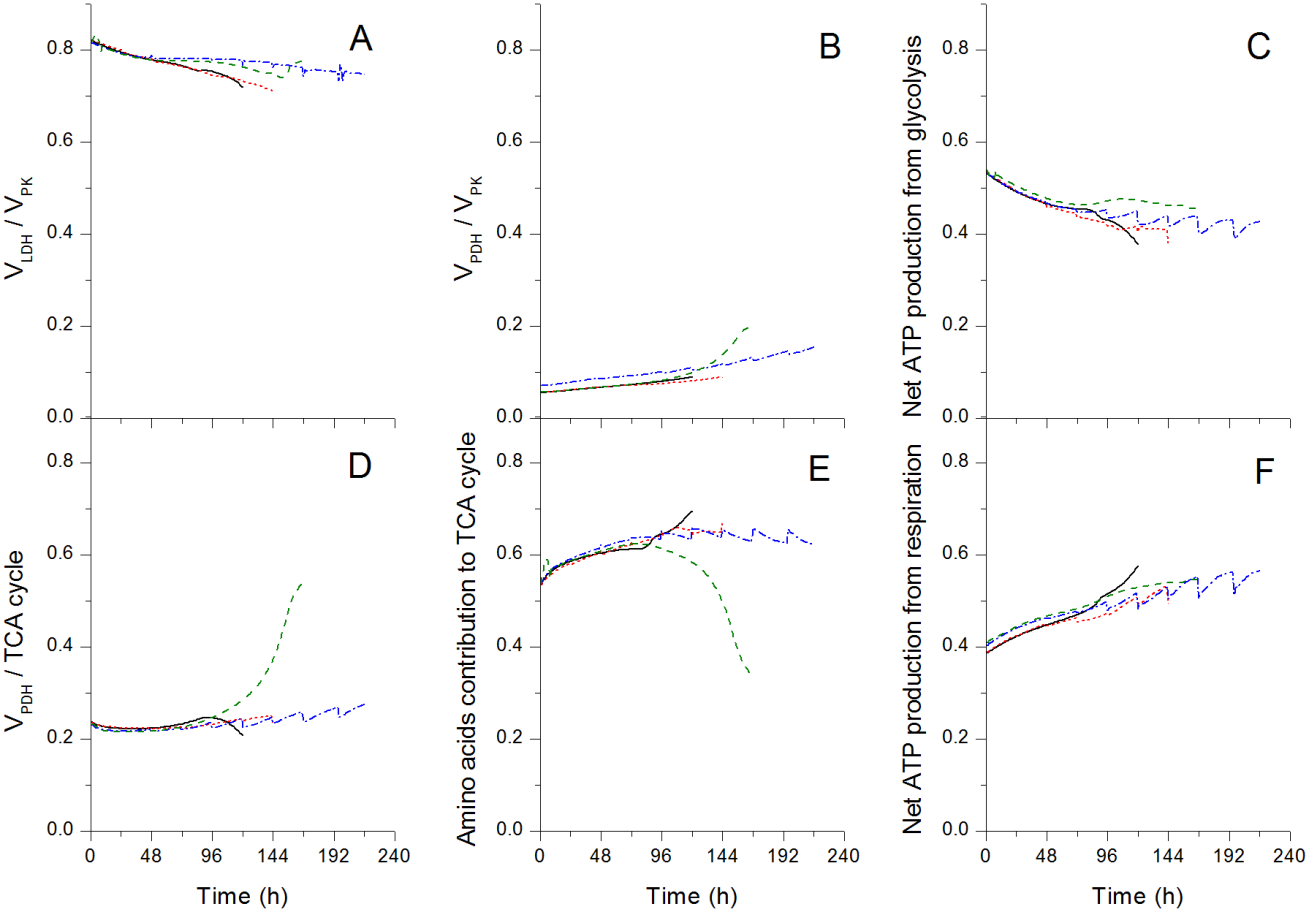
Model simulations of the metabolic ratios. Metabolic fluxes were simulated for the same conditions than in Fig 2, legend is the same as in Fig 7. Formulation of TCA cycle flux and net ATP production are the same as in Fig 7. Amino acids contribution to the TCA cycle is the ratio of the sum of the fluxes from amino acids feeding the TCA cycle (*V*_*GLDH*_+*V*_*AlaTA*_+5*V_*AAtoSUC*_) to the sum of all the fluxes feeding the TCA cycle, as defined in Fig 7. The net ATP production from glycolysis is the ratio of the sum of the ATP-producing rate from glycolysis (*V*_*HK*_+*V*_*PFK*_) and the net ATP production rate. The net ATP production from respiration is the ratio of 2*2.5*V_resp_ (or 2 moles equivalent of NADH) to the net ATP production rate.

To further investigate the behaviour of the cells looking at biological markers, the carbon flow distribution through the metabolic network was also analysed by model simulations (Fig 8 for key metabolic ratios, and S9 Table for numerical values and 95% confidence intervals at 48 and 96 h). Similarly than for most fluxes (Fig 7), flux ratios are closely similar for all culture conditions until 96 h, which corresponds to late exponential phase in batch and mid-exponential in early fed-batch operation. Then, the behaviours differ for the two batches with PowerCHO-2, while all flux ratios exhibit quasi steady-states with similar values in both fed-batch cultures. Interestingly, PowerCHO-2 batch shows opposite behaviour of that in Biogro-CHO medium from 96 h. We recall the Biogro-CHO batch reached maximum cell density at ~ 96 h compared to ~ 120 h in PowerCHO-2 batch, while cell growth extended to more than 120 h in both fed-batches (Fig 4B and 4H). Indeed, from 96 h in the PowerCHO-2 batch culture the ratio of pyruvate dehydrogenase (*V*_*PDH*_)-to-glycolytic flux (*V*_*PK*_) and the *V*_*PDH*_ contribution to the TCA cycle both suddenly increase until the end of the culture (Fig 8B and 8D respectively), while amino acids flux to the TCA cycle suddenly decreases (Fig 8E). These punctual metabolic shifts coincide with glutamine depletion observed in this PowerCHO-2 batch culture. Of interest, the lactate production-to-glucose consumption rates ratio (*V*_*LDH*_/*V*_*PK*_, Fig 8A) exhibits an enhanced stability, also in that culture compared to the batch Biogro-CHO culture. Moreover, the simulated ATP turnover rate, either in glycolysis (*V*_*HK*_+*V*_*PFK*_, Fig 8C) or from the oxidative phosphorylation (*V*_*resp*_, Fig 8F), shows a higher stability in the PowerCHO-2 batch culture. The effect of re-feeding essential medium components in fed-batch cultures clearly show stabilizing the cell metabolic efficiency for all flux ratios analysed (Fig 8), exhibiting steady-state trend for the whole culture duration regardless of the use of the Biogro-CHO or PowerCHO-2 media. Therefore, although each individual flux shows to evolve with culture time (Fig 7), the cell metabolic robustness at maintaining stable carbon flux distribution seems enhanced, including the ATP turnover rate which determines a cell global efficiency. We have previously showed that such stable cell energetic state also determines cell productive capacity [29]; thus agreeing with the model predictions of similar production capacity in all the cases under study. Specifically, the specific growth rates and mAb production rates are adequately simulated.

Our results show a robust metabolism for this cell line, when in batch mode, with little differences in the flux distribution even at the end of the experimental phase. Similar results in regards to this flux distribution was observed by Naderi et al. [25] concerning the absence of differences between exponential and stationary phases for viable cells in batch cultures. In contrast, some reports indicate that stationary phase exhibits lower glycolytic fluxes coupled with a TCA activity similar to [16] or greater [17] than the TCA cycle activity measured during the exponential phase. In our model, however, simulations were stopped rapidly in the stationary phase because of the criteria of 95% cell viability imposed. Those results from literature might have been observed in this work if the model structure would have allowed extending the simulation further at low cell viability.

When comparing batch and fed-batch-modes, model simulations are in agreement with previous work of Dorka et al. (2009) who also used a dynamic model for hybridoma cells to compare culture modes showing a similar flux distribution for both modes. However, other work performing a MFA study on NSO cells [51] showed a significantly different flux distribution comparing fed-batch to batch modes. Authors reported that either the flux distribution and the flux ratios changed when the cells switched from a lactate production mode to a lactate consumption mode, a phenomenon that has not been clearly observed in this work neither experimentally nor from model simulations, and which may explain a different conclusion in our work compared to Muluktula et al. [51]. Taking all of the above, this *in silico* dynamic metabolic flux distribution analysis thus suggests that the CHO cell line studied has a highly regulated metabolism.

### Amino acids metabolism limits growth in the latter phases of the culture

The CHO cells under study exhibit an inefficient glucose metabolism with more than 75% of glycolysis resulting in lactate production, which leads to lactate accumulation an thus growth inhibition (< 58.5 mM lactate [52]). Around only 7% of the glycolytic flux is directed toward the TCA cycle via the pyruvate dehydrogenase (*V*_*PDH*_) enzyme, while another fraction of the glycolytic flux is directed toward alanine production (*V*_*AlaTA*_) (~10%) and pyruvate carboxylase (*V*_*PC*_) (less than 5%). The inefficient use of glucose for ATP production is a well-known characteristic of CHO cells [38], converting most of the available glucose into lactate obtaining ATP from an aerobic glycolysis process. Glycolysis normally accounts for roughly 45% of the net ATP production, cell respiration being responsible for the remaining production.

Since most of medium glucose is not processed via the *V*_*PDH*_ enzyme, the TCA cycle is largely fed from amino acids metabolism (*V*_*AlaTA*_+*V*_*GLDH*_+5*V_*AAtoSUC*_), indeed accounting for 60±1 to 62±1% after 48 h depending on the culture, compared to only 22±1% for the pyruvate dehydrogenase. The major contribution of glutamine and asparagine to TCA feeding in CHO cells was reported previously [53]. Surprisingly in our case, the glutamate dehydrogenase flux (*V*_*GLDH*_) was negligible; most of the glutamate integrating the TCA cycle via alanine transferase (*V*_*AlaTA*_). Unlike *V*_*GLDH*_, *V*_*AlaTA*_ pathway does not produce directly ammonia, a metabolite known to inhibit cell growth and viability as well as recombinant proteins glycosylation pattern [7]. However, the cells do not consume extracellular glutamate, and the conversion of glutamine to glutamate via *V*_*GlnT*_, of asparagine to aspartate via *V*_*ASN*_, which can be further transformed to glutamate via aspartate transferase, both produce ammonia as a by-product. While the cells manage to maintain an efficient amino acids metabolism in regards to ammonia secretion, the use of amino acids to fuel the TCA cycle still produces undesired metabolites compared to the use of glycolysis. In our case for fed-batch culture, the inhibition term of growth equation in regards to lactate went from 0.99 initially to 0.89 using Biogro-CHO, and from 0.99 initially to 0.86 using PowerCHO-2. Concerning ammonia, however, its inhibition term in the growth equation changed from 0.957 in both fed-batch cultures initially to 0.372 for the fed-batch culture using Biogro-CHO, and 0.278 for the fed-batch culture using PowerCHO-2 after 120 h. The accumulation of ammonia, more than the accumulation of lactate, may explain the slow growth observed in the latter stages in both fed-batch cultures, and the absence of improvement of the mAb final titer compared to the batch cultures regardless of the culture medium used. Moreover, the inhibition factor linking growth rate to ammonia concentration was previously identified as a sensitive parameter (see Table 1), highlighting and agreeing with the negative effect of ammonia. Since our model does not simulate cell death mechanisms, it was then impossible to determine if ammonia is inhibiting cell growth or promoting cell death. However, from our simulation results, it is obvious that ammonia affects negatively the net cell growth rate, which is the effective sum of both growth and death rate. The choice of an inhibition mechanism may thus seem appropriate of being eventually considered.

**Table 1:** Sensitivity analysis for the selected parameters.

Parameters	Mean of the elementary effect	Standard deviation of the elementary effect
V_maxgrowth_	4.6	6.9
V_maxPK_	1.7	4.1
V_rmaxAK_	1.6	3.6
V_maxPGK_	1.4	3.5
V_maxresp_	1.4	3.6
V_fmaxAK_	1.3	2.9
V_maxHK_	1.3	3.3
V_maxATPase_	1.2	4.7
K_mATP_	1.1	4.0
K_mADP/ATP_	1.1	2.6
K_mNADH_	0.9	3.1
_$K_{dNH_{4}}{}_{growth}$_	0.9	3.4
K_mG6P_	0.9	3.4
α_AMP/ATP_	0.8	2.5
V_fmaxGlnT_	0.7	3.1
β_AMP/ATP_	0.6	2.5
V_maxleak_	0.6	1.6
V_fmaxPGI_	0.6	2.1
K_aAMP/ATP_	0.6	1.7
K_dG6P_	0.6	1.7

## Conclusion

In this work, we have further developed, accounting for the regulation of glycolysis, and adapted a kinetic metabolic model previously described for other CHO cell lines. The model was challenged with experimental data from two different culture media used in batch and fed-batch cultures. In such model, the initial conditions and the feed composition are the only inputs required to perform culture simulations. A single set of kinetic parameter values allowed describing CHO cell nutritional and metabolic behaviours under batch and fed-batch cultures using two different culture media (Biogro-CHO and PowerCHO-2). To the best of our knowledge, it is the first time a metabolic model that includes such a wide array of intracellular concentrations is used to compare culture media and fed-batch strategies for CHO cells. Given its flexible dynamic structure, while the model is cell line-specific, our results suggest that it is not culture medium-specific. In addition to the concentration profiles, given the dynamic nature of the model, we performed a dynamic metabolic flux analysis from model simulation that was in agreement with literature. Model simulations suggest that the cell line used in this study (CHO-DXB11) has a robust metabolism, showing little changes in terms of flux distribution throughout nearly all of the exponential and plateau phase. That metabolism was maintained regardless the medium or the culture mode for most of the culture duration. The fed-batch mode showed stabilizing cell metabolism for an extended period of time. However, sustained amino acids metabolism in fed-batch cultures led to important ammonia accumulation, which may have limited growth while resulting, however, in higher mAb production. We thus believe such model can be further used as an *in silico* platform and challenged for the identification of optimized medium composition and fed-batch culture strategies, guiding and accelerating bioprocess development steps.

## Supporting Information

S1 Fig Experimental and model simulation results for intracellular nucleotides. Same conditions and symbols than in Fig 2 applied.
(TIF)

S2 Fig Experimental and model simulation results for intracellular metabolites. Same conditions and symbols than in Fig 2 applied.
(TIF)

S3 Fig Experimental and model simulation results for extracellular amino acid. Same conditions and symbols than in Fig 2 applied.
(TIF)

S1 Table Reactions of the metabolic model. All units are in millimole. Conversion factors for biomass and mAb are 2.93x10^-3^mmol.10^-6^ cells and 9.17x10^-3^ mmol.mg^-1^ respectively.
(DOCX)

S2 Table Kinetic rate equations. (DOCX)

S3 Table State variables and initial conditions. (DOCX)

S4 Table Parameter values. Parameter values for all parameters, including non-optimized parameters, and the boundary of their confidence interval (95%), when it applied. All the data available was used for parameter estimation (four cultures).
(DOCX)

S5 Table Parameter values for the 20 most sensitive parameters for different media and culture modes. Parameter values and confidence intervals (95%) for the same conditions than in Fig 5.
(DOCX)

S6 Table Parameter values for the most sensitive parameters for different growth phases. Parameter values and confidence intervals (95%) for the same conditions than in Fig 6.
(DOCX)

S7 Table Metabolic fluxes at 48 and 96 h (Batch cultures). Metabolic fluxes (all in mmol.10^-6^cells.h^-1^) and their confidence intervals at 48 h and 96 h for the same conditions than in Fig 7 (Batch cultures only). Definition for ammonia production, TCA cycle and net ATP production are the same than in Fig 7.
(DOCX)

S8 Table Metabolic fluxes at 48 and 96 h (Fed-batch cultures). Metabolic fluxes (all in mmol.10^-6^cells.h^-1^) and their confidence intervals at 48 h and 96 h for the same conditions than in Fig 7 (Fed-batch cultures only). Definition for ammonia production, TCA cycle and net ATP production are the same than in Fig 7.
(DOCX)

S9 Table Metabolic flux ratios at 48 and 96 h. Metabolic ratios (all in mmol.10^-6^cells.h^-1^) and their confidence intervals at 48 h and 96 h for the same conditions than in Fig 7. Definition for ammonia production, TCA cycle and net ATP production are the same than in Fig 7.
(DOCX)
