# Supplementary figures and images for: A Single Dynamic Metabolic Model Can Describe mAb Producing CHO Cell Batch and Fed-Batch Cultures on Different Culture Media
Source: PLoS One. 2015 Sep 2;10(9):e0136815. doi: 10.1371/journal.pone.0136815 (PMC4558054; doi:10.1371/journal.pone.0136815)

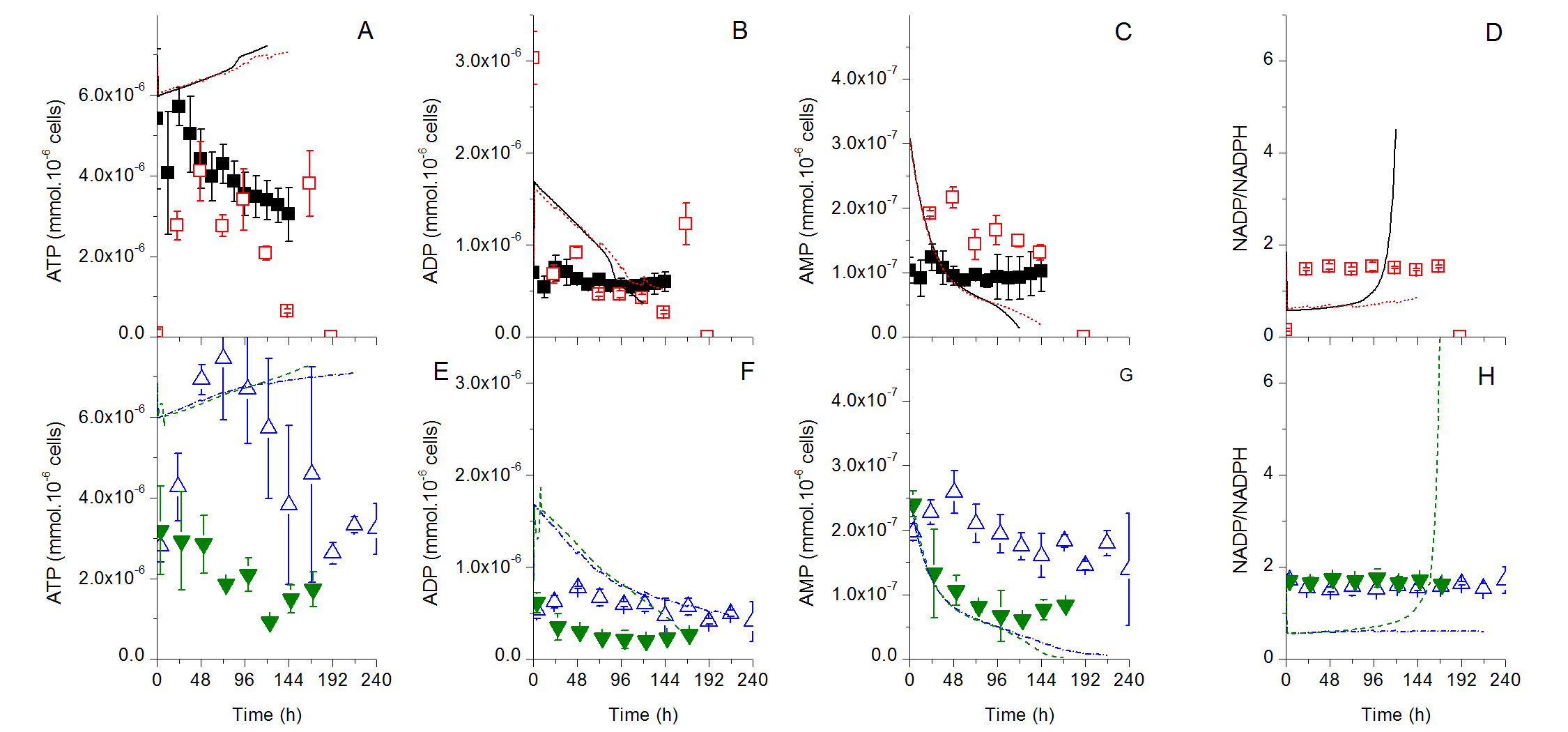

Supplement: S1 Fig — Same conditions and symbols than in Fig 2 applied. (TIF) [file pone.0136815.s001.tif]

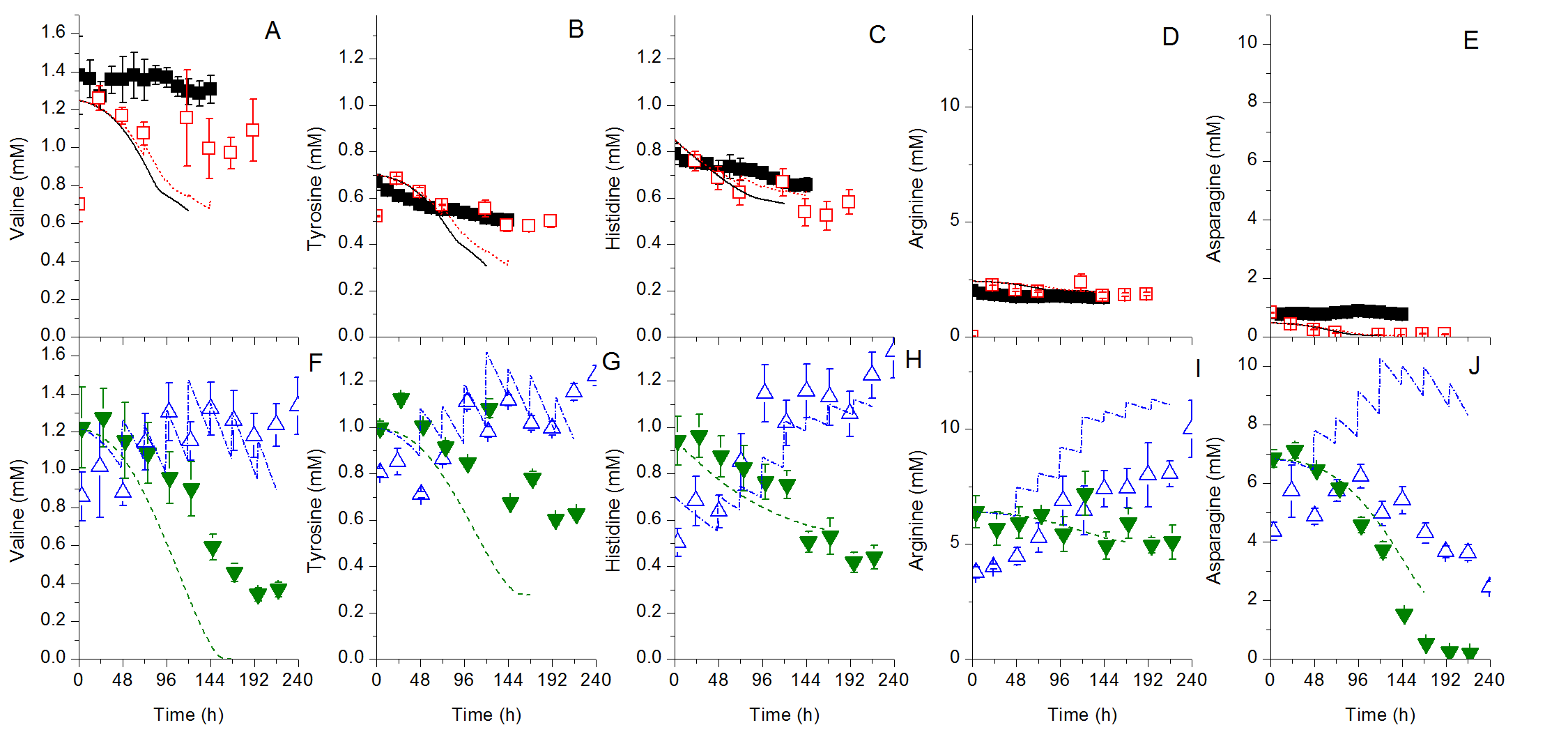

Supplement: S2 Fig — Same conditions and symbols than in Fig 2 applied. (TIF) [file pone.0136815.s002.tif]

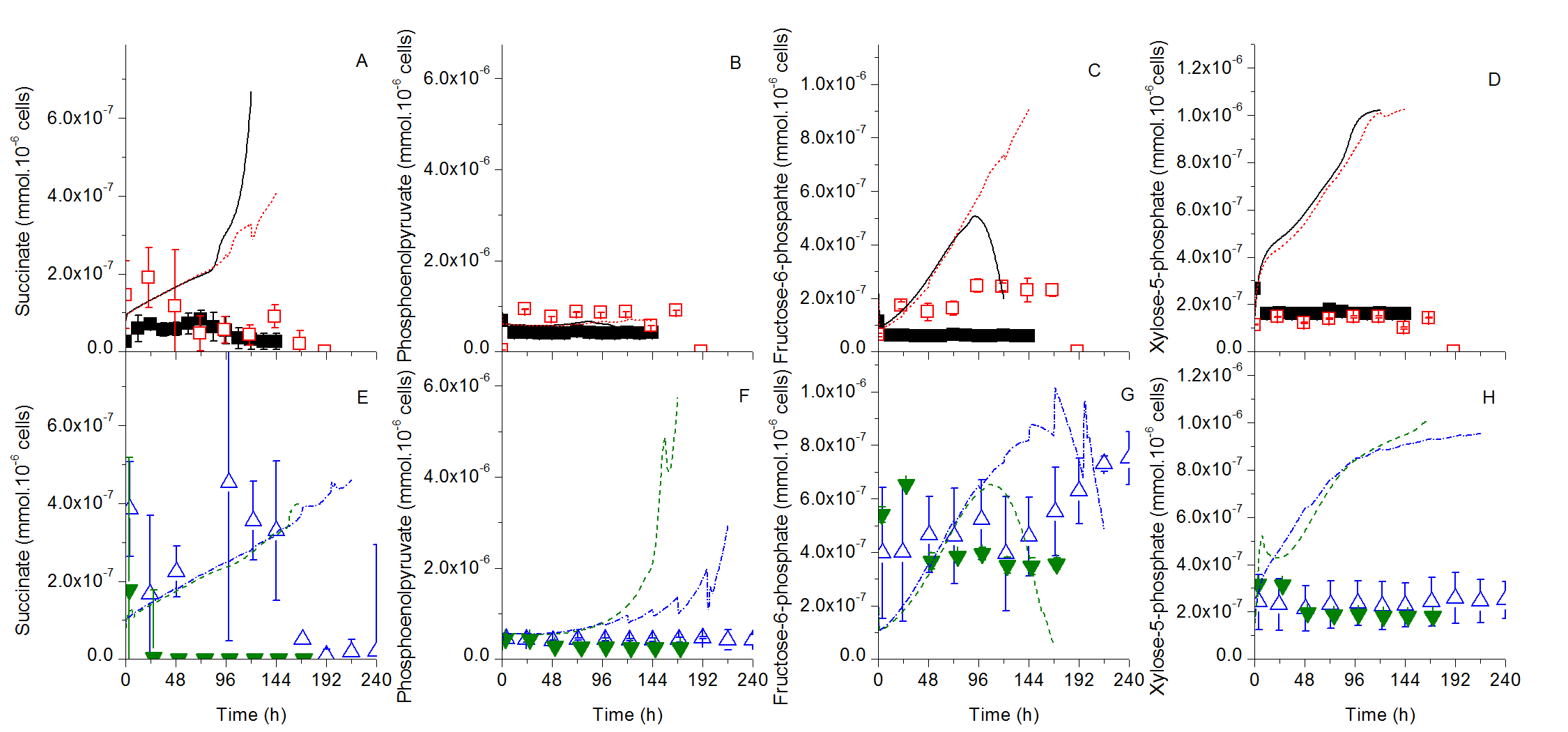

Supplement: S3 Fig — Same conditions and symbols than in Fig 2 applied. (TIF) [file pone.0136815.s003.tif]
