# Supplementary material for: A Single Dynamic Metabolic Model Can Describe mAb Producing CHO Cell Batch and Fed-Batch Cultures on Different Culture Media
Source: PLoS One. 2015 Sep 2;10(9):e0136815. doi: 10.1371/journal.pone.0136815 (PMC4558054; doi:10.1371/journal.pone.0136815)
Supplement: S1 Table — All units are in millimole. Conversion factors for biomass and mAb are 2.93x10^-3^mmol.10-6 cells and 9.17x10^-3^ mmol.mg-1 respectively. (DOCX) [file pone.0136815.s004.docx]

| No | Name | Abbreviation | Stoichiometry |
| --- | --- | --- | --- |
| 1 | Hexokinase | V_HK_ | $EGLC+ATP\xrightarrow{}G6P+ADP$ |
| 2 | Phosphoglucose isomerase | V_PGI_ | $G6P\xleftrightarrow{}F6P$ |
| 3 | Phosphofructokinase | V_PFK_ | $F6P+ATP\xrightarrow{}2GAP+ADP$ |
| 4 | Phosphoglycerate kinase | V_PGK_ | $GAP+ADP+NAD^{+}\xrightarrow{}PEP+ATP+NADH$ |
| 5 | Pyruvate kinase | V_PK_ | $PEP+ADP\xrightarrow{}PYR+ATP$ |
| 6 | Lactate dehydrogenase | V_LDH_ | $PYR+NADH\xleftrightarrow{}LAC+NAD^{+}$ |
| 7 | Glucose-6-phosphate dehydrogenase | V_G6PDH_ | $G6P+2NAPD^{+}\xrightarrow{}R5P+2NADPH$ |
| 8 | Ribulose-5-phosphate epimerase | V_EP_ | $R5P\xrightarrow{}X5P$ |
| 9 | Transketolase | V_TK_ | $R5P+2X5P\xrightarrow{}2F6P+GAP$ |
| 10 | Pyruvate dehydrogenase | V_PDH_ | $PYR+NAD^{+}\xrightarrow{}ACCOA+NADH$ |
| 11 | Citrate synthase | V_CS_ | $ACCOA+OXA\xrightarrow{}CIT$ |
| 12 | Aconitase/isocitrate dehydrogenase | V_CITS_ | $CIT+NAD^{+}\xrightarrow{}AKG+NADH$ |
| 13 | Alpha ketoglutarate dehydrogenase | V_AKGDH_ | $AKG+NAD^{+}\xrightarrow{}SUC+NADH$ |
| 14 | Succinate dehydrogenase | V_SDH_ | $\begin{matrix} SUC+ADP+2/3NAD^{+} \\ \xrightarrow{}MAD+ATP+2/3NADH \end{matrix}$ |
| 15 | Malate dehydrogenase | V_MLD_ | $MAL+NAD^{+}\xrightarrow{}OXA+NADH$ |
| 16 | Pyruvate carboxylase | V_PC_ | $PYR+ATP\xrightarrow{}OXA+ADP$ |
| 17 | Malic enzyme | V_ME_ | $MAL+NAD^{+}\xrightarrow{}PYR+NADH$ |
| 18 | Glutamine synthetase | V_GlnT_ | $EGLN+ADP\xleftrightarrow{}GLU+ATP+NH_{4}^{+}$ |
| 19 | Glutamate dehydrogenase | V_GLDH_ | $GLU+NAD^{+}\xleftrightarrow{}AKG+NH_{4}^{+}+NADH$ |
| 20 | Alanine aminotranferase | V_AlaTA_ | $PYR+GLU\xleftrightarrow{}AKG+ALA$ |
| 21 | Asparaginase | V_ASN_ | $ASN\xrightarrow{}ASP+NH_{4}^{+}$ |
| 22 | Aspartate aminotransferase | V_ASTA_ | $ASP+AKG\xrightarrow{}OXA+GLU$ |
| 23 | Amino acids transamination | V_AAtoSUC_ | $\begin{matrix} LYS+ILE+LEU+VAL +TYR+7AKG+ATP \\ +9NAD+2NADP\xleftrightarrow{}4GLU+3SUC+MAL \\ +8ACCOA+ADP+9NADH+2NADPH \end{matrix}$ |
| 24 | Histidine/arginine transamination | V_HISARGTA_ | $HIS+ARG+AKG\xrightarrow{}4GLU+NH_{4}^{+}$ |
| 25 | Glutamate transport | V_GluT_ | $GLU\xrightarrow{}EGLU$ |
| 26 | Serine dehydratase | V_SDHH_ | $SER\xrightarrow{}PYR+NH_{4}^{+}$ |
| 27 | ATPase | V_ATPase_ | $ATP\xrightarrow{}ADP$ |
| 28 | NADPH oxidase | V_NADPHox_ | $NADPH\xrightarrow{}NADP^{+}$ |
| 29 | Respiration | V_resp_ | $\begin{matrix} 2NADH+O_{2}+(2P/Oratio)\mathrm{ADP} \\ \xrightarrow{}2NAD^{+}+2ATP \end{matrix}$ |
| 30 | Leak | V_leak_ | $2NADH\xrightarrow{}2NAD^{+}$ |
| 31 | Adenylate kinase | V_AK_ | $ATP+AMP\xleftrightarrow{}2ADP$ |
| 32 | Nucleotide synthesis | V_PPRibP_ | $\begin{matrix} 0.6R5P+2EGLN+2ASP+GLY+2ATP \\ \xrightarrow{}2ADP+AMP+2GLY+2MAL \end{matrix}$ |
| 33 | Biomass synthesis | V_growth_ | $\begin{matrix} 0.06ALA+0.04ARG+0.04ASN+0.03ASP \\ +0.02GLN+0.04GLU+0.06GLY+0.02HIS \\ +0.09ILE+0.06LYS+0.01MET+0.02PHE \\ +0.03PRO+0.05SER+0.04THR+0.02TYR \\ +0.04VAL+3.78ATP+0.03G6P+0.03R5P \\ +0.09CIT\xrightarrow{}X+3.78ADP \end{matrix}$ |
| 34 | mAb synthesis | V_mAb_ | $\begin{matrix} 0.06ALA+0.02ARG+0.05ASN+0.04ASP+0.04GLN \\ +0.05GLU+0.07GLY+0.02HIS+0.10ILE+0.06LYS \\ +0.01MET+0.04PHE+0.07PRO+0.11SER+0.11THR \\ +0.03TYR+0.09VAL+4ATP\xrightarrow{}mAb+4ADP \end{matrix}$ |
