# Supplementary material for: A Single Dynamic Metabolic Model Can Describe mAb Producing CHO Cell Batch and Fed-Batch Cultures on Different Culture Media
Source: PLoS One. 2015 Sep 2;10(9):e0136815. doi: 10.1371/journal.pone.0136815 (PMC4558054; doi:10.1371/journal.pone.0136815)
Supplement: S2 Table — (DOCX) [file pone.0136815.s005.docx]

| No | Equation |
| --- | --- |
| 1 | $\begin{matrix} V_{HK}=V_{\max HK}\frac{EGLC\left(1+\frac{\beta_{AMP/ATP}\mathrm{AMP}/ATP}{\alpha_{AMP/ATP}K_{A_{AMP/ATP}}}\right)}{K_{mEGLC}\left(1+\frac{\mathrm{AMP}/ATP}{K_{A_{AMP/ATP}}}\right)+EGLC\left(1+\frac{\mathrm{AMP}/ATP}{\alpha_{AMP/ATP}K_{A_{AMP/ATP}}}\right)} \\ *\frac{\mathrm{ATP}/ADP}{K_{mATP/ADP}+ATP/ADP}*\frac{EGLC}{K_{mEGLC}+EGLC}*\frac{K_{dG6P}}{K_{dG6P}+G6P} \end{matrix}$ |
| 2 | $V_{PGI}=V_{\mathrm{fmaxPGI}}*\frac{G6P}{K_{mG6P}+G6P}*\frac{K_{dPEP}}{K_{dPEP}+PEP}-V_{\mathrm{rmaxPGI}}*\frac{F6P}{K_{mG6P}+F6P}$ |
| 3 | $\begin{matrix} V_{PFK}=V_{\mathrm{maxPF}K}\frac{F6P\left(1+\frac{\beta_{AMP/ATP}\mathrm{AMP}/ATP}{\alpha_{AMP/ATP}K_{A_{AMP/ATP}}}\right)}{K_{mF6P}\left(1+\frac{\mathrm{AMP}/ATP}{K_{A_{AMP/ATP}}}\right)+F6P\left(1+\frac{\mathrm{AMP}/ATP}{\alpha_{AMP/ATP}K_{A_{AMP/ATP}}}\right)} \\ *\frac{\mathrm{ATP}/ADP}{K_{mATP/ADP}+ATP/ADP}*\frac{K_{dLAC}}{K_{dLAC}+LAC} \end{matrix}$ |
| 4 | $V_{PGK}=V_{\max PGK}*\frac{GAP}{K_{mG6P}+GAP}*\frac{ADP/ATP}{K_{mADP/ATP}+ADP/ATP}*\frac{NAD/NADH}{K_{mNAD/NADH}+NAD/NADH}$ |
| 5 |  |
| 6 |  |
| 7 | $V_{G6PDH}=V_{\max G6PDH}*\frac{G6P}{K_{mG6P}+G6P}*\frac{NADP/NADPH}{K_{mNADP/NADPH}+NADP/NADPH}$ |
| 8 | $V_{EP}=V_{\max EP}*\frac{R5P}{K_{mR5P}+R5P}$ |
| 9 | $V_{\mathrm{TK}}=V_{\mathrm{maxTK}}*\frac{R5P}{K_{mR5P}+R5P}*\frac{X5P}{K_{mX5P}+X5P}$ |
| 10 | $V_{PDH}=V_{\max PDH}*\frac{PYR}{K_{mPYR}+PYR}*\frac{NAD/NADH}{K_{mNAD/NADH}+NAD/NADH}$ |
| 11 | $V_{CS}=V_{\mathrm{maxCS}}*\frac{OXA}{K_{mOXA}+OXA}*\frac{ACCOA}{K_{mACCOA}+ACCOA}$ |
| 12 | $V_{CITS}=V_{\mathrm{maxCITS}}*\frac{CIT}{K_{mCIT}+CIT}*\frac{NAD/NADH}{K_{mNAD/NADH}+NAD/NADH}$ |
| 13 | $V_{\mathrm{AKGD}H}=V_{\mathrm{maxAKG}DH}*\frac{AKG}{K_{mAKG}+AKG}*\frac{NAD/NADH}{K_{mNAD/NADH}+NAD/NADH}$ |
| 14 | $V_{SUC}=V_{\mathrm{maxSUC}}*\frac{SUC}{K_{mSUC}+SUC}*\frac{NAD/NADH}{K_{mNAD/NADH}+NAD/NADH}*\frac{ADP/ATP}{K_{mADP/ATP}+ADP/ATP}$ |
| 15 | $V_{MLD}=V_{\max MLD}*\frac{MAL}{K_{mMAL}+MAL}*\frac{NAD/NADH}{K_{mNAD/NADH}+NAD/NADH}$ |
| 16 | $V_{PC}=V_{\max PC}*\frac{PYR}{K_{mPYR}+PYR}*\frac{ADP/ATP}{K_{mADP/ATP}+ADP/ATP}$ |
| 17 |  |
| 18 | $\begin{matrix} \mathrm{V}_{\mathrm{GlnT}}=V_{\mathrm{fmaxGln}T}*\frac{EGLN}{K_{mEGLN}+EGLN}*\frac{ATP/ADP}{K_{mATP/ADP}+ATP/ADP} \\ -V_{\mathrm{rmaxGlnT}}*\frac{GLU}{K_{mGLU}+GLU}*\frac{ADP/ATP}{K_{mADP/ATP}+ADP/ATP}*\frac{NH_{4}}{K_{mNH_{4}}+NH_{4}} \end{matrix}$ |
| 19 | $\begin{matrix} \mathrm{V}_{\mathrm{GLDH}}=V_{\mathrm{fmax}GLDH}*\frac{GLU}{K_{mGLU}+GLU}*\frac{NAD/NADH}{K_{mNAD/NADH}+NAD/NADH} \\ -V_{\mathrm{rmaxGLDH}}*\frac{AKG}{K_{mAKG}+AKG}*\frac{NADH/NAD}{K_{mNADH/NAD}+NADH/NAD}*\frac{NH_{4}}{K_{mNH_{4}}+NH_{4}} \end{matrix}$ |
| 20 | $\begin{matrix} V_{AlaTA}=V_{\mathrm{fmaxA}laTA}*\frac{GLU}{K_{mGLU}+GLU}*\frac{PYR}{K_{mPYR}+PYR} \\ -V_{\mathrm{rmax}AlaTA}*\frac{ALA}{K_{mALA}+ALA}*\frac{AKG}{K_{mAKG}+AKG}*\frac{K_{dEGLN}}{K_{dEGLN}+EGLN} \end{matrix}$ |
| 21 | $V_{ASN}=V_{\mathrm{maxASN}}*\frac{ASN}{K_{mASN}+ASN}$ |
| 22 | $V_{ASTA}=V_{\mathrm{maxAS}TA}*\frac{AKG}{K_{mAKG}+AKG}*\frac{ASP}{K_{mASP}+ASP}$ |
| 23 | $\begin{matrix} V_{AAtoSUC}=V_{\mathrm{maxAAtoSUC}}*\frac{LYS}{K_{mLYS}+LYS}*\frac{ILE}{K_{mILE}+ILE}*\frac{AKG}{K_{mAKG}+AKG}*\frac{VAL}{K_{mVAL}+VAL} \\ *\frac{NAD/NADH}{K_{mNAD/NADH}+NAD/NADH}*\frac{NAPD/NAPDH}{K_{mNADP/NAPDH}+NAPD/NAPDH}*\frac{ATP/ADP}{K_{mATP/ADP}+ATP/ADP} \end{matrix}$ |
| 24 | $\mathrm{V}_{\mathrm{HISARG}TA}=V_{\mathrm{maxHISARGTA}}*\frac{HIS}{K_{mHIS}+HIS}*\frac{ARG}{K_{mARG}+ARG}*\frac{LEU}{K_{mLEU}+LEU}*\frac{AKG}{K_{mAKG}+AKG}$ |
| 25 | $V_{GluT}=V_{\mathrm{fmaxGluT}}*\frac{GLU}{K_{mGLU}+GLU}-V_{\mathrm{rmaxGluT}}*\frac{EGLU}{K_{mEGLU}+EGLU}$ |
| 26 | $V_{SDHH}=V_{\mathrm{maxSDHH}}*\frac{SER}{K_{mSER}+SER}$ |
| 27 | $V_{ATPase}=V_{\max ATPase}*\frac{ATP}{K_{mATP}+ATP}$ |
| 28 | $V_{NADPHox}=V_{\max NADPHox}*\frac{NADPH}{K_{mNADPH}+NADPH}$ |
| 29 | $V_{resp}=V_{\mathrm{maxresp}}*\frac{ADP/ATP}{K_{mADP/ATP}+ADP/ATP}*\frac{NADH}{K_{mNADH}+NADH}$ |
| 30 | $V_{leak}=V_{\mathrm{maxleak}}*\frac{NADH}{K_{mNADH}+NADH}$ |
| 31 | $V_{AK}=V_{\mathrm{fmaxAK}}*\frac{AMP}{K_{mAMP}+AMP}*\frac{ATP}{K_{mATP}+ATP}-V_{\mathrm{rmax}AK}*\frac{ADP}{K_{mADP}+ADP}$ |
| 32 | $\begin{matrix} V_{PPRibP}=V_{\max PPRibP}*\frac{R5P}{K_{mR5P}+R5P}*\frac{EGLN}{K_{mEGLN}+EGLN}*\frac{ASP}{K_{mASP}+ASP}*\frac{GLY}{K_{mGLY}+GLY} \\ *\frac{ATP}{K_{mATP}+ATP} \end{matrix}$ |
| 33 |  |
| 34 |  |
