# Supplementary material for: A Single Dynamic Metabolic Model Can Describe mAb Producing CHO Cell Batch and Fed-Batch Cultures on Different Culture Media
Source: PLoS One. 2015 Sep 2;10(9):e0136815. doi: 10.1371/journal.pone.0136815 (PMC4558054; doi:10.1371/journal.pone.0136815)
Supplement: S3 Table — (DOCX) [file pone.0136815.s006.docx]

| Components | Unit | Biogro-CHO | | PowerCHO-2 | |
| --- | --- | --- | --- | --- | --- |
|  |  | Batch | Fed-batch | Batch | Fed-batch |
| ACCOA | mmol.10^-6^ cell | 1.4E-7 | 1.4E-7 | 1.4E-7 | 1.4E-7 |
| ADP | mmol.10^-6^ | 6.7E-7 | 6.7E-7 | 6.7E-7 | 6.7E-7 |
| AKG | mmol.10^-6^ | 1.0E-7 | 1.0E-7 | 1.0E-7 | 1.0E-7 |
| ALA | mM | 9.6E-1 | 9.6E-1 | 7.3E-1 | 7.3E-1 |
| AMP | mmol.10^-6^ cell | 3.1E-7 | 3.1E-7 | 2.5E-7 | 2.5E-7 |
| ARG | mM | 2.4 | 2.4 | 6.4 | 6.4 |
| ASN | mM | 4.8E-1 | 4.8E-1 | 6.9 | 6.9 |
| ASP | mM | 2.0 | 2.0 | 9.5 | 9.5 |
| ATP | mmol.10^-6^ cell | 7.0E-6 | 2.2E-6 | 3.5E-6 | 3.5E-6 |
| CIT | mmol.10^-6^ cell | 1.0E-6 | 1.0E-6 | 1.0E-6 | 1.0E-6 |
| CYS | mM | 1.0E-1 | 1.0E-1 | 2.1E-2 | 2.1E-2 |
| EGLC | mM | 26 | 10 | 43 | 41 |
| EGLN | mM | 3.7 | 2.4 | 6.0 | 6.0 |
| EGLU | mM | 8.0E-1 | 8.0E-1 | 8.0E-1 | 8.0E-1 |
| F6P | mmol.10^-6^ cell | 2.2E-7 | 2.2E-7 | 2.2E-7 | 2.2E-7 |
| G6P | mmol.10^-6^ cell | 1.6E-7 | 1.6E-7 | 1.6E-7 | 1.6E-7 |
| GAP | mmol.10^-6^ cell | 4.0E-7 | 4.0E-7 | 4.0E-7 | 4.0E-7 |
| GLU | mmol.10^-6^ cell | 2.2E-4 | 2.2E-4 | 2.2E-4 | 2.2E-4 |
| GLY | mM | 7.8E-1 | 7.8E-1 | 5.8 | 5.8 |
| HIS | mM | 8.5E-1 | 8.5E-1 | 9.4E-1 | 7.0E-1 |
| ILE | mM | 2.2 | 2.2 | 7.4 | 5.0 |
| LAC | mM | 7.5E-1 | 7.5E-1 | 7.5E-1 | 7.5E-1 |
| LYS | mM | 2.1 | 2.1 | 5.7 | 5.7 |
| mAb | mg.L^-1^ | 0.0 | 0.0 | 0.0 | 0.0 |
| MAL | mmol.10^-6^ cell | 1.5E-6 | 1.5E-6 | 1.5E-6 | 1.5E-6 |
| MET | mM | 4.3E-1 | 4.3E-1 | 1.7 | 1.1 |
| NAD | mmol.10^-6^ cell | 6.9E-7 | 6.9E-7 | 6.9E-7 | 6.9E-7 |
| NADH | mmol.10^-6^ cell | 8.5E-7 | 8.5E-7 | 8.5E-7 | 8.5E-7 |
| NADP | mmol.10^-6^ cell | 3.5E-7 | 3.5E-7 | 2.7E-7 | 2.7E-7 |
| NADPH | mmol.10^-6^ cell | 2.3E-7 | 2.3E-7 | 1.5E-7 | 1.5E-7 |
| NH4 | mM | 2.0E-1 | 2.0E-1 | 2.0E-1 | 2.0E-1 |
| OXA | mmol.10^-6^ cell | 1.0E-6 | 1.0E-6 | 1.0E-6 | 1.0E-6 |
| PEP | mmol.10^-6^ cell | 8.8E-7 | 8.8E-7 | 1.8E-7 | 1.8E-7 |
| PHE | mM | 1.1 | 1.1 | 1.0 | 6.1E-1 |
| PRO | mM | 6.5E-1 | 6.5E-1 | 2.3 | 1.4 |
| PYR | mmol.10^-6^ cell | 2.0E-6 | 2.0E-6 | 6.5E-6 | 6.5E-6 |
| R5P | mmol.10^-6^ cell | 5.4E-8 | 5.4E-8 | 5.4E-8 | 5.4E-8 |
| SER | mM | 8.3E-1 | 8.3E-1 | 8.9 | 5.8 |
| SUC | mmol.10^-6^ cell | 8.0E-8 | 8.0E-8 | 8.0E-8 | 8.0E-8 |
| THR | mM | 1.1 | 1.1 | 1.1 | 1.1 |
| TYR | mM | 1.3 | 1.3 | 1.2 | 1.2 |
| VAL | mM | 7.0E-1 | 7.0E-1 | 1.0 | 1.0 |
