# Supplementary material for: A Single Dynamic Metabolic Model Can Describe mAb Producing CHO Cell Batch and Fed-Batch Cultures on Different Culture Media
Source: PLoS One. 2015 Sep 2;10(9):e0136815. doi: 10.1371/journal.pone.0136815 (PMC4558054; doi:10.1371/journal.pone.0136815)
Supplement: S4 Table — Parameter values for all parameters, including non-optimized parameters, and the boundary of their confidence interval (95%), when it applied. All the data available was used for parameter estimation (four cultures). (DOCX) [file pone.0136815.s007.docx]

| Parameter | Units | Value | Confidence region |
| --- | --- | --- | --- |
| __ | - | 4.7E-01 | [4.4E-01 5.1E-01] |
| __ | _-_ | 4.1E+00 | - |
| __ | _-_ | 1.046E+01 | [1.045E+01 1.046E+01] |
| __ | _-_ | 1.7E+00 | - |
| __ | _-_ | 9.0E-02 | [8.8E-02 9.3E-02] |
| __ | mmol.10^-6^cells | 4.9E-06 | - |
| __ | mmol.10^-6^cells | 7.4E-04 | - |
| __ | mM | 3.5E+00 | - |
| __ | mmol.10^-6^cells | 1.710841E-08 | [1.710839E-08 1.710842E-08] |
| __ | mM | 6.3E+00 | - |
| __ | mmol.10^-6^cells | 3.7E-07 | - |
| __ | mmol.10^-6^cells | 4.5E-07 | - |
| __ | mM | 1.7E+02 | - |
| __ | mM | 4.44E+00 | [4.13E+00 4.74E+00] |
| __ | mmol.10^-6^cells | 8.6E-07 | - |
| __ | mmol.10^-6^cells | 7.2E-07 | - |
| __ | mmol.10^-6^cells | 1.1E-06 | - |
| __ | mM | 1.8E+01 | - |
| __ | mmol.10^-6^cells | 2.4E-09 | - |
| __ | mM | 7.3E-02 | - |
| __ | mM | 4.0E-02 | - |
| __ | mM | 1.2E+00 | - |
| __ | mmol.10^-6^cells | 1.56E-05 | [1.55E-05 1.57E-05] |
| __ | mmol.10^-6^cells | 1.6E-07 | - |
| __ | mM | 2.0E+01 | - |
| __ | mM | 2.3E+00 | - |
| __ | mM | 6.6E+00 | - |
| __ | mmol.10^-6^cells | 2.6E-06 | - |
| __ | mmol.10^-6^cells | 2.866E-08 | [2.865E-08 2.866E-08] |
| __ | mmol.10^-6^cells | 7.8E-07 | - |
| __ | mmol.10^-6^cells | 7.5E-05 | - |
| __ | mmol.10^-6^cells | 5.0E-02 | - |
| __ | mM | 5.8E-01 | - |
| __ | mM | 3.8E-02 | - |
| __ | mM | 2.9E+00 | - |
| __ | mM | 0.- | - |
| __ | mM | 9.8E-02 | - |
| __ | mmol.10^-6^cells | 2.7E-06 | - |
| __ | mmol.10^-6^cells | 2.6E-06 | [2.4E-06 2.8E-06] |
| __ | mmol.10^-6^cells | 8.4E-08 | - |
| __ | mM | 1.1E+00 | - |
| __ | mmol.10^-6^cells | 2.2E-07 | - |
| __ | mmol.10^-6^cells | 1.7E-07 | - |
| __ | mmol.10^-6^cells | 3.9E-06 | - |
| __ | mmol.10^-6^cells | 7.0E-08 | - |
| __ | mM | 2.2E-01 | - |
| __ | mmol.10^-6^cells | 2.3E-07 | - |
| __ | mM | 4.8E-02 | - |
| __ | mM | 1.- | - |
| __ | mmol.10^-6^cells | 1.1E-08 | - |
| __ | - | 2.1E+00 | - |
| __ | mmol.10^-6^cells.h^-1^ | 1.8E-08 | [1.7E-08 1.9E-08] |
| __ | mmol.10^-6^cells.h^-1^ | 1.2E-08 | [1.1E-08 1.2E-08] |
| __ | mmol.10^-6^cells.h^-1^ | 2.6E-04 | - |
| __ | mmol.10^-6^cells.h^-1^ | 1.7E-04 | - |
| __ | mmol.10^-6^cells.h^-1^ | 2.4E-04 | - |
| __ | mmol.10^-6^cells.h^-1^ | 6.2E-06 | - |
| __ | mmol.10^-6^cells.h^-1^ | 7.8E-05 | - |
| __ | mmol.10^-6^cells.h^-1^ | 9.14E-04 | [9.09E-04 9.13E-04] |
| __ | mmol.10^-6^cells.h^-1^ | 3.9E-05 | - |
| __ | mmol.10^-6^cells.h^-1^ | 8.8E-05 | - |
| __ | mmol.10^-6^cells.h^-1^ | 1.3E-05 | - |
| __ | mmol.10^-6^cells.h^-1^ | 1.5E-05 | - |
| __ | mmol.10^-6^cells.h^-1^ | 1.2E-06 | - |
| __ | mmol.10^-6^cells.h^-1^ | 6.3E-05 | - |
| __ | mmol.10^-6^cells.h^-1^ | 1.27E-04 | [1.11E-04 1.42E-04] |
| __ | mmol.10^-6^cells.h^-1^ | 1.9E-05 | - |
| __ | mmol.10^-6^cells.h^-1^ | 1.8E-06 | - |
| __ | mmol.10^-6^cells.h^-1^ | 2.5E-06 | - |
| __ | h^-1^ | 6.6E-02 | [6.5E-02 6.7E-02] |
| __ | mmol.10^-6^cells.h^-1^ | 1.9E-05 | - |
| __ | mmol.10^-6^cells.h^-1^ | 6.6E-04 | [6.2E-04 6.9E-04] |
| __ | mmol.10^-6^cells.h^-1^ | 1.7E-03 | - |
| __ | mmol.10^-6^cells.h^-1^ | 2.4E-04 | - |
| __ | mmol.10^-6^cells.h^-1^ | 2.9E-05 | [2.8E-05 3.0E-05] |
| __ | mmol.10^-6^cells.h^-1^ | 1.3E-04 | - |
| __ | mmol.10^-6^cells.h^-1^ | 2.2E-04 | - |
| __ | mmol.10^-6^cells.h^-1^ | 1.3E-05 | - |
| __ | mmol.10^-6^cells.h^-1^ | 9.5E-05 | - |
| __ | mmol.10^-6^cells.h^-1^ | 1.4E-05 | - |
| __ | mmol.10^-6^cells.h^-1^ | 2.1E-04 | - |
| __ | mmol.10^-6^cells.h^-1^ | 7.1E-05 | - |
| __ | mmol.10^-6^cells.h^-1^ | 1.5E-03 | - |
| __ | mmol.10^-6^cells.h^-1^ | 7.6E-04 | [6.9E-04 8.3E-04] |
| __ | mmol.10^-6^cells.h^-1^ | 3.5E-04 | - |
| __ | mmol.10^-6^cells.h^-1^ | 1.065E-03 | [1.055E-03 1.075E-03] |
| __ | mmol.10^-6^cells.h^-1^ | 1.376E-03 | [1.373E-03 1.378E-03] |
| __ | mmol.10^-6^cells.h^-1^ | 2.6E-09 | - |
| __ | mmol.10^-6^cells.h^-1^ | 1.40E-03 | [1.29E-03 1.50E-03] |
| __ | mmol.10^-6^cells.h^-1^ | 4.0E-04 | - |
| __ | mmol.10^-6^cells.h^-1^ | 8.5E-06 | - |
| __ | mmol.10^-6^cells.h^-1^ | 2.3E-05 | - |
| __ | mM | 7.6E-02 | - |
| __ | mM | 1.5E-02 | - |
| __ | mM | 4.2E-03 | - |
| __ | mM | 1.0E-02 | - |
| __ | mM | 1.4E-07 | - |
| __ | mmol.10^-6^cells | 3.5E-09 | - |
| __ | mM | 1.0E-03 | - |
| __ | mM | 1.4E-03 | - |
|  | mmol.10^-6^cells | 9.9E-13 | - |
|  | mmol.10^-6^cells | 1.7E-06 | - |
|  | mM | 1.0E-02 | - |
|  | mM | 1.7E-02 | - |
|  | mM | 1.0E-02 | - |
|  | mM | 2.7E-02 | - |
|  | mM | 3.5E-02 | - |
|  | mM | 1.6E-02 | - |
|  | mM | 1.0E-02 | - |
|  | mmol.10^-6^cells | 1.1E-10 | - |
|  | mM | 1.1E-03 | - |
|  | mM | 9.6E-03 | - |
|  | mM | 1.2E-02 | - |
|  | mM | 1.3E-02 | - |
|  | mM | 1.3E-01 | - |
|  | mM | 6.2E-02 | - |
|  | mM | 1.4E-06 | - |
|  | mM | 5.6E-02 | - |
|  | mM | 1.1E-07 | - |
|  | mM | 1.0E-03 | - |
|  | mM | 1.3E-03 | - |
|  | mmol.10^-6^cells | 2.2E-01 | - |
|  | mM | 5.0E-02 |  |
|  | mM | 5.7E-02 | - |
|  | mM | 8.3E-02 | - |
|  | mM | 0.- | - |
|  | mM | 5.0E-02 | - |
|  | mM | 8.0E-02 | - |
|  | mM | 7.1E-02 | - |
|  | mM | 1.1E-02 | - |
|  | mM | 1.0E-03 | - |
|  | mM | 6.6E-02 | - |
|  | mM | 9.8E-02 | - |
|  | mM | 7.4E-02 | - |
|  | - | 5.69423E-01 | [5.69422E-01 5.69425E-01] |
|  | - | 9.1E-01 | - |
|  | - | 4.8E-02 | - |
|  | - | 8.3E+00 | - |
|  | - | 2.0E+00- | - |
