# Supplementary material for: A Single Dynamic Metabolic Model Can Describe mAb Producing CHO Cell Batch and Fed-Batch Cultures on Different Culture Media
Source: PLoS One. 2015 Sep 2;10(9):e0136815. doi: 10.1371/journal.pone.0136815 (PMC4558054; doi:10.1371/journal.pone.0136815)
Supplement: S5 Table — Parameter values and confidence intervals (95%) for the same conditions than in Fig 5. (DOCX) [file pone.0136815.s008.docx]

| Parameters | All cultures | | Fed-batch cultures | | Batch cultures | | Biogro-CHO | | PowerCHO-2 | |
| --- | --- | --- | --- | --- | --- | --- | --- | --- | --- | --- |
|  | Values | Confidence intervals | Values | Confidence intervals | Values | Confidence intervals | Values | Confidence intervals | Values | Confidence intervals |
| __ | 6.6E-2 | 8E-4 | 6.6E-2 | 9E-9 | 6.6E-2 | 4E-4 | 6.6E-2 | 3E-3 | 7.2E-2 | 1E-2 |
| __ | 1.4E-3 | 3E-6 | 1.4E-3 | 2E-4 | 1.4E-3 | 1E-5 | 1.4E-3 | 3E-5 | 1.4E-3 | 2E-5 |
| __ | 1.2E-8 | 7E-10 | 1.2E-8 | 1E-8 | 1.2E-8 | 2E-9 | 1.2E-8 | 4E-9 | 1.1E-8 | 7E-11 |
| __ | 1.1E-3 | 1E-5 | 1.1E-3 | 1E-4 | 1.1E-3 | 6E-8 | 1.1E-3 | 2E-5 | 9.9E-4 | 5E-5 |
| __ | 1.4E-3 | 1E-4 | 1.4E-3 | 2E-3 | 1.5E-3 | 2E-4 | 1.4E-3 | 7E-5 | 1.7E-3 | 1E-5 |
| __ | 1.8E-8 | 9E-10 | 1.8E-8 | 2E-8 | 1.8E-8 | 4E-9 | 1.8E-8 | 7E-10 | 1.8E-8 | 4E-9 |
| __ | 6.6E-4 | 4E-5 | 6.6E-4 | 3E-4 | 6.6E-4 | 3E-4 | 6.6E-4 | 9E-10 | 6.4E-4 | 6E-4 |
| $V_{\max ATPase}$ | 9.1E-4 | 4E-6 | 9.1E-4 | 2E-4 | 8.3E-4 | 1E-4 | 9.1E-4 | 3E-4 | 8.1E-4 | 7E-5 |
| __ | 1.6E-5 | 1E-7 | 1.6E-5 | 1E-5 | 1.6E-5 | 2E-6 | 1.6E-5 | 6E-6 | 1.8E-5 | 4E-6 |
| __ | 5.7E-1 | 1E-6 | 5.7E-1 | 1E-6 | 5.7E-1 | 1E-2 | 5.7E-1 | 1E-1 | 6.7E-1 | 1E-2 |
| __ | 2.6E-6 | 2E-7 | 2.6E-6 | 4E-6 | 2.6E-6 | 3E-7 | 2.6E-6 | 1E-7 | 2.3E-6 | 9E-8 |
| __ | 4.4E+0 | 3E-1 | 4.4E+0 | 6E-1 | 4.3E+0 | 4E-2 | 4.4E+0 | 2E+0 | 3.5E+0 | 1E+0 |
| __ | 2.9E-8 | 6E-12 | 2.9E-8 | 1E-8 | 2.8E-8 | 1E-9 | 2.9E-8 | 9E-9 | 3.6E-8 | 1E-10 |
| __ | 4.7E-1 | 3E-2 | 4.7E-1 | 4E-1 | 4.7E-1 | 1E-1 | 4.7E-1 | 9E-1 | 5.4E-1 | 4E-1 |
| __ | 1.3E-4 | 2E-5 | 1.3E-4 | 3E-5 | 1.3E-4 | 2E-5 | 1.3E-4 | 3E-5 | 1.2E-4 | 6E-6 |
| __ | 1.0E+1 | 2E-3 | 1.0E+1 | 8E-1 | 1.0E+1 | 2E+0 | 1.0E+1 | 9E-6 | 1.1E+1 | 2E+0 |
| __ | 2.9E-5 | 1E-6 | 2.9E-5 | 4E-5 | 2.9E-5 | 2E-6 | 2.9E-5 | 1E-5 | 2.6E-5 | 1E-5 |
| __ | 7.6E-4 | 7E-5 | 7.6E-4 | 2E-4 | 7.6E-4 | 3E-6 | 7.6E-4 | 1E-4 | 6.5E-4 | 2E-6 |
| __ | 9.0E-2 | 3E-3 | 9.0E-2 | 7E-2 | 9.1E-2 | 3E-4 | 9.0E-2 | 2E-1 | 1.5E-1 | 1E-1 |
| __ | 1.7E-8 | 1E-14 | 1.7E-8 | 1E-14 | 1.7E-8 | 2E-9 | 1.7E-8 | 4E-11 | 1.7E-8 | 1E-8 |
