# Supplementary material for: A Single Dynamic Metabolic Model Can Describe mAb Producing CHO Cell Batch and Fed-Batch Cultures on Different Culture Media
Source: PLoS One. 2015 Sep 2;10(9):e0136815. doi: 10.1371/journal.pone.0136815 (PMC4558054; doi:10.1371/journal.pone.0136815)
Supplement: S6 Table — Parameter values and confidence intervals (95%) for the same conditions than in Fig 6. (DOCX) [file pone.0136815.s009.docx]

| Parameters | Exponential growth (0 to 72 h) | | End exponential to plateau (>72 h) | |
| --- | --- | --- | --- | --- |
|  | Mean value | Confidence interval (95 %) | Mean value | Confidence interval (95 %) |
| __ | 6.6E-02 | 3E-03 | 6.6E-02 | 3E-03 |
| __ | 1.4E-03 | 4E-06 | 1.4E-03 | 3E-07 |
| __ | 1.2E-08 | 2E-09 | 1.2E-08 | 3E-10 |
| __ | 1.1E-03 | 4E-05 | 1.1E-03 | 4E-06 |
| __ | 1.4E-03 | 2E-05 | 1.4E-03 | 3E-07 |
| __ | 1.8E-08 | 1E-09 | 1.8E-08 | 6E-10 |
| __ | 6.6E-04 | 3E-08 | 6.6E-04 | 2E-06 |
| $V_{\max ATPase}$ | 9.1E-04 | 2E-04 | 9.2E-04 | 3E-06 |
| __ | 1.6E-05 | 7E-07 | 1.6E-05 | 1E-07 |
| __ | 5.7E-01 | 7E-03 | 5.7E-01 | 7E-04 |
| __ | 2.6E-06 | 5E-09 | 2.6E-06 | 4E-08 |
| __ | 4.4E+00 | 8E-02 | 4.4E+00 | 8E-05 |
| __ | 2.9E-08 | 8E-13 | 2.6E-08 | 9E-10 |
| __ | 4.7E-01 | 4E-02 | 4.7E-01 | 3E-04 |
| __ | 1.3E-04 | 1E-06 | 1.3E-04 | 1E-08 |
| __ | 1.0E+01 | 1E+00 | 1.0E+01 | 1E-03 |
| __ | 2.9E-05 | 2E-06 | 2.9E-05 | 2E-09 |
| __ | 7.6E-04 | 1E-04 | 7.6E-04 | 2E-06 |
| __ | 9.0E-02 | 1E-03 | 1.1E-01 | 2E-04 |
| __ | 1.7E-08 | 6E-18 | 1.7E-08 | 2E-11 |
