# Supplementary material for: A Single Dynamic Metabolic Model Can Describe mAb Producing CHO Cell Batch and Fed-Batch Cultures on Different Culture Media
Source: PLoS One. 2015 Sep 2;10(9):e0136815. doi: 10.1371/journal.pone.0136815 (PMC4558054; doi:10.1371/journal.pone.0136815)
Supplement: S7 Table — Metabolic fluxes (all in mmol.10-6cells.h-1) and their confidence intervals at 48 h and 96 h for the same conditions than in Fig 7 (Batch cultures only). Definition for ammonia production, TCA cycle and net ATP production are the same than in Fig 7. (DOCX) [file pone.0136815.s010.docx]

| Metabolic fluxes | Batch, Biogro-CHO | | | | Batch, PowerCHO-2 | | | |
| --- | --- | --- | --- | --- | --- | --- | --- | --- |
|  | Mean value | | Confidence interval | | Mean value | | Confidence interval | |
|  | 48 h | 96 h | 48 h | 96 h | 48 h | 96 h | 48 h | 96 h |
| *V_HK_* | 2.1E-04 | 7.9E-05 | 1.6E-05 | 2.1E-05 | 2.2E-04 | 1.4E-04 | 1.6E-05 | 1.4E-05 |
| *V_LDH_* | 1.9E-04 | 6.6E-05 | 1.5E-05 | 1.3E-05 | 1.9E-04 | 1.3E-04 | 1.6E-05 | 1.2E-05 |
| *V_G6PDH_* | 3.6E-06 | 3.3E-06 | 3.0E-07 | 7.2E-07 | 3.7E-06 | 3.9E-06 | 2.5E-07 | 2.2E-07 |
| *V_GlnT_* | 4.5E-05 | 1.4E-06 | 5.8E-06 | 1.1E-06 | 5.9E-05 | 1.3E-05 | 8.6E-06 | 8.2E-06 |
| *V_AlaTA_* | 3.6E-05 | 1.5E-05 | 3.2E-06 | 2.1E-06 | 3.8E-05 | 2.8E-05 | 3.7E-06 | 3.1E-06 |
| Ammonia production | 5.5E-05 | 2.1E-06 | 5.8E-06 | 1.2E-06 | 7.7E-05 | 3.0E-05 | 8.6E-06 | 8.3E-06 |
| TCA cycle | 7.4E-05 | 3.1E-05 | 7.1E-06 | 4.0E-06 | 7.7E-05 | 5.5E-05 | 8.1E-06 | 6.9E-06 |
| *V_PDH_* | 1.6E-05 | 7.5E-06 | 1.6E-06 | 1.3E-06 | 1.7E-05 | 1.4E-05 | 1.8E-06 | 1.7E-06 |
| Net ATP production | 1.1E-03 | 4.3E-04 | 7.0E-05 | 5.5E-05 | 1.1E-03 | 7.4E-04 | 9.4E-05 | 9.4E-05 |
