# Supplementary material for: A Single Dynamic Metabolic Model Can Describe mAb Producing CHO Cell Batch and Fed-Batch Cultures on Different Culture Media
Source: PLoS One. 2015 Sep 2;10(9):e0136815. doi: 10.1371/journal.pone.0136815 (PMC4558054; doi:10.1371/journal.pone.0136815)
Supplement: S9 Table — Metabolic ratios (all in mmol.10-6cells.h-1) and their confidence intervals at 48 h and 96 h for the same conditions than in Fig 7. Definition for ammonia production, TCA cycle and net ATP production are the same than in Fig 7. (DOCX) [file pone.0136815.s012.docx]

| Metabolic flux | Fed-batch, Biogro-CHO | | | | Fed-batch, PowerCHO-2 | | | | Fed-batch, Biogro-CHO | | | | Fed-batch, PowerCHO-2 | | | |
| --- | --- | --- | --- | --- | --- | --- | --- | --- | --- | --- | --- | --- | --- | --- | --- | --- |
|  | Mean value | | Confidence interval | | Mean value | | Confidence interval | | Mean value | | Confidence interval | | Mean value | | Confidence interval | |
|  | 48 h | 96 h | 48 h | 96 h | 48 h | 96 h | 48 h | 96 h | 48 h | 96 h | 48 h | 96 h | 48 h | 96 h | 48 h | 96 h |
| *V_LDH_*/*V_PK_* | 0.78 | 0.74 | 0.03 | 0.03 | 0.78 | 0.74 | 0.02 | 0.03 | 0.79 | 0.78 | 0.03 | 0.03 | 0.78 | 0.77 | 0.03 | 0.03 |
| *V_PDH_*/*V_PK_* | 0.07 | 0.08 | 0.01 | 0.01 | 0.07 | 0.05 | 0.01 | 0.01 | 0.07 | 0.08 | 0.01 | 0.01 | 0.07 | 0.08 | 0.01 | 0.01 |
| Net ATP production from glycolysis | 0.47 | 0.41 | 0.03 | 0.03 | 0.46 | 0.42 | 0.03 | 0.02 | 0.45 | 0.42 | 0.03 | 0.02 | 0.46 | 0.45 | 0.03 | 0.03 |
| V_PDH_ /TCA cycle | 0.22 | 0.24 | 0.005 | 0.01 | 0.22 | 0.16 | 0.01 | 0.01 | 0.22 | 0.22 | 0.004 | 0.01 | 0.22 | 0.25 | 0.005 | 0.01 |
| Amino acids contribution toTCA cycle | 0.60 | 0.67 | 0.01 | 0.02 | 0.61 | 0.65 | 0.01 | 0.01 | 0.62 | 0.65 | 0.01 | 0.01 | 0.61 | 0.61 | 0.01 | 0.01 |
| Net ATP production from respiration | 0.45 | 0.54 | 0.03 | 0.03 | 0.44 | 0.47 | 0.03 | 0.03 | 0.45 | 0.46 | 0.03 | 0.03 | 0.45 | 0.49 | 0.03 | 0.03 |
